# Atypical cellular responses mediated by intracellular constitutive active TrkB (NTRK2) kinase domains and a solely intracellular NTRK2-fusion oncogene

**DOI:** 10.1038/s41417-024-00809-0

**Published:** 2024-07-22

**Authors:** Rohini Gupta, Melanie Dittmeier, Gisela Wohlleben, Vera Nickl, Thorsten Bischler, Vanessa Luzak, Vanessa Wegat, Dennis Doll, Annemarie Sodmann, Elena Bady, Georg Langlhofer, Britta Wachter, Steven Havlicek, Jahnve Gupta, Evi Horn, Patrick Lüningschrör, Carmen Villmann, Bülent Polat, Jörg Wischhusen, Camelia M. Monoranu, Jochen Kuper, Robert Blum

**Affiliations:** 1 Department of Neurology, University Hospital Würzburg, Würzburg, Germany; 2 Institute of Clinical Neurobiology, University Hospital Würzburg, Würzburg, Germany; 3 Department of Radiation Oncology, University of Würzburg, Würzburg, Germany; 4 Department of Neurosurgery, Section Experimental Neurosurgery, University Hospital Würzburg, Würzburg, Germany; 5 Core Unit Systems Medicine, University of Würzburg, Würzburg, Germany; 6 Department of Obstetrics and Gynecology, University Hospital Würzburg, Würzburg, Germany; 7 Department of Neuropathology, Institute of Pathology, University of Würzburg, Würzburg, Germany; 8 Rudolf Virchow Center for Experimental Biomedicine, Institute for Structural Biology, University of Würzburg, Würzburg, Germany; 9 Present Address: Ludwig-Maximilians-Universität München, Biomedizinisches Zentrum, Planegg, Germany; 10 Present Address: Fraunhofer-Institut für Grenzflächen- und Bioverfahrenstechnik IGB, Bio- Elektro- und Chemokatalyse BioCat, Straubing, Germany; 11 Present Address: Institute of Pathology, University Medical Center Hamburg-Eppendorf, Hamburg, Germany; 12 Present Address: Neurona Therapeutics, 170 Harbor Way, South San Francisco, CA USA

**Keywords:** Cell biology, Molecular biology

## Abstract

Trk (*NTRK*) receptor and *NTRK* gene fusions are oncogenic drivers of a wide variety of tumors. Although Trk receptors are typically activated at the cell surface, signaling of constitutive active Trk and diverse intracellular *NTRK* fusion oncogenes is barely investigated. Here, we show that a high intracellular abundance is sufficient for neurotrophin-independent, constitutive activation of TrkB kinase domains. In HEK293 cells, constitutive active TrkB kinase and an intracellular NTRK2-fusion oncogene (SQSTM1-NTRK2) reduced actin filopodia dynamics, phosphorylated FAK, and altered the cell morphology. Atypical cellular responses could be mimicked with the intracellular kinase domain, which did not activate the Trk-associated MAPK/ERK pathway. In glioblastoma-like U87MG cells, expression of TrkB or SQSTM1-NTRK2 reduced cell motility and caused drastic changes in the transcriptome. Clinically approved Trk inhibitors or mutating Y^705^ in the kinase domain, blocked the cellular effects and transcriptome changes. Atypical signaling was also seen for TrkA and TrkC. Moreover, hallmarks of atypical pTrk kinase were found in biopsies of Nestin-positive glioblastoma. Therefore, we suggest Western blot-like immunoassay screening of NTRK-related (brain) tumor biopsies to identify patients with atypical panTrk or phosphoTrk signals. Such patients could be candidates for treatment with NTRK inhibitors such as Larotrectinhib or Entrectinhib.

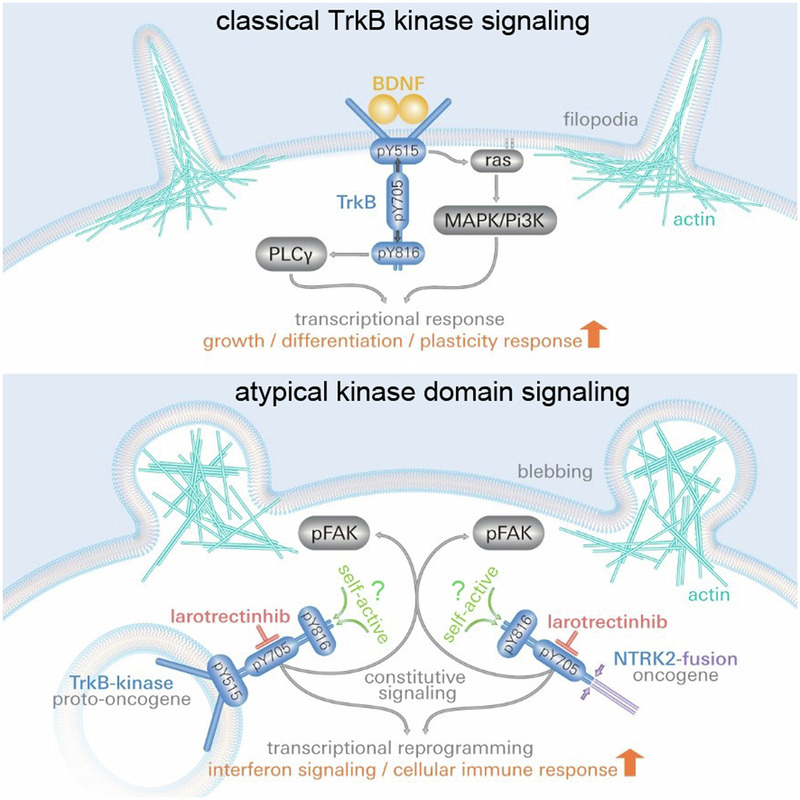

## Introduction

Tropomyosin receptor kinase (Trk) receptors belong to a family of membrane-bound receptor tyrosine kinases. Trk was originally discovered as an oncogenic driver in human colon carcinoma [1]. The three NTRK genes *NTRK1*, *NTRK2*, and *NTRK3* encode neurotrophin receptors TrkA, TrkB, and TrkC, respectively [2, 3]. Trk receptors play important roles in the development and physiology of the nervous system, neuronal differentiation, and synaptic communication [4–6]. Trk receptors are typically activated by a family of secreted neurotrophins. Binding of neurotrophins to the extracellular domains of Trk induces receptor dimerization and subsequent trans-autophosphorylation [7–11]. After autophosphorylation, the kinases recruit many signaling pathways, including Shc/Ras/Erk, Pi3K/Akt, mTOR, or PLCγ/Ca^2+^ signaling [6–8,12]. Trk receptors can also be transactivated in the absence of neurotrophins [13–17] and isolated intracellular domains of TrkA and TrkB can undergo autophosphorylation [18, 19]. This in vitro autophosphorylation and activation of kinase domains of TrkA and TrkB is concentration-dependent and is enhanced by dimerization [18]. The kinase domain of TrkA has a higher catalytic efficiency than the kinase domain of TrkB. Data suggest that this is sequence-dependent and caused by a higher tendency to self-dimerize [18]. Furthermore, TrkA has been shown to become constitutive active at intracellular sites when glycosylation and maturation of the receptor domain are artificially blocked [20].

Trk receptors and pathological *NTRK* gene fusions are protumorigenic in various adult and pediatric tumor types [12, 21]. Moreover, Trk kinase overexpression and genomic amplification of NTRK have been observed in a range of human cancers [12, 22]. Constitutive TrkB and TrkC receptor signaling has been associated with tumorigenesis in glioblastoma [23, 24] and TrkB and TrkC signaling is also abundant in glioblastoma, the most common type of brain tumor in adults [25]. Fusions of *NTRK1*, *NTRK2*, and *NTRK3* genes belong to the genomic landscape of diverse types of gliomas [26–30]. Due to these results, the recent WHO classification of CNS tumors, has listed NTRK proteins as diagnostic markers for infant-type hemispheric glioma [28].

To target tumorigenic Trk kinase domains, small-molecule Trk inhibitors have been developed [31]. These tumor-agnostic drugs, e.g. Larotrectinib or Entrectinib, are modern pharmaceuticals for precision oncology and show good response rates to NTRK fusion-positive cancers [12,32–36]. However, detection of NTRK-related tumors is not easy, especially not in the case of brain tumors. For instance, anti-human pan Trk antibodies are in use to detect NTRK fusion tumors, but these antibodies also detect all three natural Trk kinases.

Albeit neurotrophin-dependent Trk signaling is well known, the mechanisms by which atypically activated receptors affect cell function are not fully conclusive [6, 12]. For instance, there are protumorigenic, kinase-active *NTRK* fusion proteins that lack the extracellular ligand-binding domain, the transmembrane, as well as the juxtamembrane domains, and even the phosphorylation adapter site for the Ras/MAPK/ERK pathway, i.e. the critical protein domains required for Trk kinase dimerization and signaling [12, 27, 37]. Furthermore, immunohistochemistry of NTRK-positive tumor samples localized NTRK fusion proteins to other cellular compartments than native Trk receptors [38]. This raises the question of how intracellular Trk kinase domains become constitutive active when important protein domains for ER translocation, cell surface transport, ligand binding, dimerization, and kinase activation are missing [12]. For activation of intracellular NTRK fusion proteins, two mechanisms are plausible: (1) dimerization and transactivation are forced by the fusion domain [12, 39] or (2) abundance-dependent kinase domains undergo autoactivation and become constitutive active, as they do in vitro [18, 19] (discussed in [6]).

Here, we describe atypical constitutive signaling of the TrkB kinase and of a prototypical intracellular NTRK2-fusion oncogene. These atypical signaling effects can be blocked with clinically approved Trk inhibitors. The effect was also seen for TrkA and TrkC. Furthermore, we found underglycosylated, atypical phospho-active Trk kinase signals in single glioblastoma biopsies, but not in human brain control samples. These atypical panTrk signals can easily be detected using western blot techniques with anti-panTrk kinase and anti-phospho-Trk antibodies.

## Materials and methods

### Cloning and plasmids

All mammalian expression vectors used in this study were constructed using pcDNA3 or the lentiviral vector FuGW [40]. We referred to the cDNA sequence *Ntrk2* (*trk*B full-length – *trk*B.FL) (reference: NM_001025074 / NP001020245) for all mouse TrkB constructs. Doxycycline-inducible lentiviral expression of TrkB constructs was performed using a vector backbone based on pCW [41]. The pCW vector expresses a puromycin resistance gene cassette under the control of the ubiquitous hPGK1 promoter. All constructs used in this study are listed in Table 1. Lentiviral constructs in FuGW carry an N-terminal HA-tag between the signal peptide and the first amino acid of the mature TrkB receptor [42]. Mutants were generated by site-directed mutagenesis using synthetic oligonucleotides and Quick Change II XL mutagenesis kit (Agilent Technologies). An additional human *Ntrk2* fusion construct (SQSTM1 fused to TrkB kinase [37] was generated by gene synthesis (Eurofins).

**Table 1:** Mammalian expression vectors.

pcDNA3 vectors & identifier	LV FuGW vectors/( + IRES-Cre)	LV pCW vectors
pcDNA3-TrkB wt (#715)	pFU-HA-TrkB wt (#717) / (#901)	pCW-TrkB wt (#1029)
pcDNA3-TrkB Shc (#747)	pFU-HA-TrkB Shc (#780) / (#906)	
pcDNA3-TrkB ATP (#749)	pFU-HA-TrkB ATP (#761) / (#905)	
pcDNA3-TrkB YFF (#759)	pFU-HA-TrkB YFF (#788) / (#910)	pCW-TrkB YFF (#1030)
pcDNA3-TrkB PLCγ (#752)	pFU-HA-TrkB PLCγ (#792) / (#907)	
pcDNA3-TrkB YYF (#837)	pFU-HA-TrkB YYF (#847) / (#909)	
pcDNA3-TrkB YFY (#838)	pFU-HA-TrkB YFY (#846) / (#908)	
pcDNA3-TrkB YDY (#871)	pFU-HA-TrkB YDY (#917) / (#920)	
pcDNA3-TrkB YEY (#872)		
pcDNA3-TrkB Shc/PLCγ (#873)	pFU-HA-TrkB Shc/PLCγ (#918) / (#921)	
pcDNA3-TrkB ATP-YDY (#894)	pFU-HA-TrkB ATP-YDY (#919) / (#922)	
pcDNA3-TrkB ATP-YEY (#895)		
pcDNA3-TrkB S478A (#974)		
	pFU-TrkB-ICD (#993)	pCW-TrkB ICD (#1032)
	pFU-TrkB-myrICD (#995)	pCW-TrkB MyrICD (#1033)
pcDNA3-*SQSTM1-NTRK2* (#1020)		pCW-*SQSTM1-NTRK2* (#1035)
	pFUGW (Lois et al., 2002)	
pGFP-Actin^81^		
pcDNA3-TrkB-T1 (#161) (Rose et al., 2003)		
pcDNA3-TrkC-wt (#197)		
pcDNA3-TrkA-wt (#205)		

### Cell culture and transfections

HEK293 cells (ACC cat# 305) were grown in DMEM with high glucose, GlutaMAX (Gibco), 10% FCS, 100 units/ml penicillin, and 100 µg/ml streptomycin (Gibco). U87MG, a glioblastoma-like cell line [43] (ATCC, Cat# HTB-14) and NIH-3T3 (Cell lines Services, #400101) were cultured in the same medium. Cells were incubated at 37 °C in 5% CO_2_. For transfection, Lipofectamine 2000 (Invitrogen) was used at a ratio of 1 µg DNA per 2 µl Lipofectamine. The medium was replaced after 24 h, and expression was maintained for 30–72 h.

### Lentivirus and LV-generated TrkB-expressing U87 MG cells

Lentiviral particles were packaged in HEK293TN producer cells (SBI Biosciences) with pCMV-VSVG and pCMVΔR8.91 [44] helper plasmids. Cells were transfected with Lipofectamine 2000 (Invitrogen) in OptiMEM with 10% FCS for 12–14 h. Viral supernatants were harvested 72 h after transfection by ultracentrifugation. Viral particles were suspended in 50 mM Tris-HCl, pH 7.8), 130 mM NaCl,10 mM KCl, and 5 mM MgCl_2_ and stored at −80 °C. The viral titer was tested in HEK293 cells. The number of infectious particles was determined using serial dilutions of the viral vectors in HEK293 cells. For the transduction of U87MG cells, a multiplicity of infection (m.o.i.) of one was used. One day after transduction, the U87MG cells were cultured in the presence of 1 µg/ml puromycin.

### Cell transformation assay in NIH-3T3 cells

Cell transformation in NIH-3T3 cells was performed by testing the morphological transformation of adherent NIH-3T3 cells [45, 46]. To generate stable NIH-3T3 cells expressing TrkB constructs, 150,000 cells were infected with LV particles (see above) and were subsequently cultured for three days. Then, cells were split, seeded on 25 cm² flasks and were continuously cultured in the presence of 2 µg/ml puromycin. After two more cell passages, cells were used for the experiments. For induction of expression of TrkB constructs, cells were treated with 1 mg/ml doxycycline (Dox). For testing NIH-3T3 cell transformation, 300,000 cells were plated on 6 cm dishes and cultured for 10–12 days. Cells were then fixed with methanol (−20 °C) for 30 min and afterward labeled with a classical Giemsa stain. For immunolabeling, 50,000 cells were plated on 10 mm glass coverslips. One day after cell seeding, Trk expression was induced with Dox. For Western blotting, 150,000 cells were grown on 3.5 cm dished, treated with Dox after 24 h, and were finally harvested after 48–72 h.

### Cell migration assay

U87MG cells expressing the TrkB constructs were seeded at a density of 20,000 cells per well into a 2-well silicone insert (Ibidi, Cat# 81176), positioned in a 35 mm µ-dish (35 mm, high, Ibidi, Cat# 81156). One day after seeding, 1 mg/ml Dox was added to induce the expression of the corresponding TrkB-related constructs (Dox on). DMSO was used as the control. 24 h after Dox induction, the cell culture dishes were filled with growth medium and the silicone insert was removed. The cells were monitored using brightfield microscopy directly after removal of the insert and after 24 h to analyze cell migration. Subsequently, the cells were fixed and labeled using immunofluorescence.

The detection and quantification of cells in the acquired brightfield images were automated with a custom-written code in ImageJ (Rasband, W.S., ImageJ, U.S. National Institutes of Health, Bethesda, Maryland, USA, https://imagej.nih.gov/ij/). Images were converted to 8-bit grayscale and smoothed by replacing each pixel with the mean of its neighborhood (5-pixel radius). Cells were defined as local maxima with a prominence greater than 7.5 in the smoothened images. The area of the removed silicone insert was annotated manually in each image, and the number of cells detected within this area was quantified.

### Antibodies

Primary antibodies were used for indirect immunofluorescence labeling or Western blot analysis at the indicated dilutions (Table 2).

**Table 2:** Key resources table.

Reagent or Resource	Source	Identifier
Primary antibodies – dilution from stock		
Rabbit monoclonal anti-panTrk (C-term) – A7H6R (1:500 – 1:10,000) (anti-panTrk)	Cell Signaling	Cat# 92991; RRID: AB_2800196
Rabbit monoclonal anti-SQSTM1/p62 (D5E2) (1:1000)	Cell Signaling	Cat# 8025 RRID: AB_10859911
Rabbit monoclonal anti-pY490-TrkA (anti-pY516-TrkB) C35G9 – Shc site (1:500) (anti-pTrk-shc)	Cell Signaling	Cat# 4619; RRID: AB_10235585
Rabbit monoclonal anti-pY674/675-TrkA (anti-pY706/707–TrkB) C50F3 (1:500) (anti-pTrk-kin)	Cell Signaling	Cat# 4621; RRID: AB_916186
Rabbit monoclonal anti-pY785-TrkA (anti-pY816-TrkB) C67C8 – PLCγ site (1:500) (anti-pTrk-PLCγ)	Cell Signaling	Cat# 4168; RRID: AB_10620952
Goat polyclonal anti-TrkB (receptor domain) (1:1000) (anti-TrkB)	R&D Systems	Cat# AF1494; RRID: AB_2155264
Rabbit polyclonal anti-FAK (1:500)	Cell Signaling	Cat# 3285; RRID: AB_2269034
Rabbit polyclonal anti- pY397-FAK (1:500)	Cell Signaling	Cat# 3283; RRID: AB_2173659
Rabbit polyclonal anti-pY576/577-FAK (1:500)	Cell Signaling	Cat# 3281; RRID: AB_331079
Rabbit polyclonal anti- pY925-FAK (1:500)	Cell Signaling	Cat# 3284; RRID: AB_10831810
Rabbit monoclonal anti-44/42-MAP Kinase 137F5 (1:1000)	Cell Signaling	Cat# 4695; RRID: AB_390779
Rabbit monoclonal anti-p44/42-MAP Kinase (D13.14.4E) (1:1000)	Cell Signaling	Cat# 4370; RRID: AB_2315112
Rabbit polyclonal anti-Cofilin (1:1000)	Cell Signaling	Cat# 3312; RRID: AB_330235
Rabbit monoclonal anti-pS3-Cofilin 77G2 (1:1000)	Cell Signaling	Cat# 3313; RRID: AB_2080597
Mouse monoclonal anti-Nestin (human) 10C2 (1:1000) (Western)	Merck Millipore	Cat# MAB5326; RRID: AB_2251134
Mouse monoclonal anti-hNestin (1:2400) (IHC)	R&D Systems	Cat# MAB1259; RRID: AB_2251304
Mouse monoclonal anti-γ-Adaptin (1:1000)	BD Biosciences	Cat# 610385; RRID: AB_397768
Mouse monoclonal anti-HA.11 Clone 16B12	Covance	Cat# MMS-101P; RRID: AB_10064068
Rabbit monoclonal anti SQSTM1/p62 (D5E2)	Cell Signaling	Cat# 8025; RRID:AB_10859911
Secondary antibodies (all stored as 0.5 mg/ml)		
Donkey anti goat IgG affiniPure (H + L)-Alexa488 (1:800)	Jackson	Cat# 705-545-147; RRID: AB_2336933
Donkey anti goat affiniPure-Alexa647	Jackson	Cat# 705-605-003; RRID: AB_2340436
Donkey anti goat HRP IgG (H + L) (1:5000)	Jackson	Cat# 705-035-147; RRID: AB_2313587
Donkey anti rabbit IgG affiniPure (H + L)-Cy3-550 (1:800)	Jackson	Cat# 711-165-152; RRID: AB_2307443
Peroxidase AffiniPure Goat Anti-Rabbit IgG (H + L) (1:5000)	Jackson	Cat# 111-035-144; RRID: AB_2307391
Goat anti mouse IgG affiniPure (H + L)-Alexa488 (1:800)	Invitrogen	Cat# A11029; RRID: AB_138404
Peroxidase AffiniPure Goat Anti-Mouse IgG (H + L) (1:5000)	Jackson	Cat# 115-035-146; RRID: AB_2307392
Goat Anti-Mouse IgG1 affiniPure, Cy3-conjugated, Fc_Subclass 1-specific (human glioblastoma tissue)	Jackson	Cat# 115-165-205; RRID: AB_2338694
FACS: R-Phycoerythrin AffiniPure Goat Anti-Mouse IgG, Fcγ subclass 2a specific	Jackson	Cat# 115-115-206 RRID: AB_2338621
Chemicals, Peptides and Recombinant Proteins		
Acti-stain-670-Phalloidin	Cytoskeleton	Cat# PHDN1
Ammonium persulfate (APS)	Sigma–Aldrich	Cat# A3678
Ampicillin	Roth	Cat# K029
Aquapolymount	Polyscienes Inc.	Cat# 18606
Blotting Grade Blocker – non-fat dry milk	BioRad	Cat# 1706404
β-mercaptoethanol	Sigma	Cat# M7154
BSA (Bovine Serum Albumin)	Sigma–Aldrich	Cat# A7030
BDNF (brain-derived neurotrophic factor) – with carrier	R&D Systems	Cat# 248-BDB
Bromophenol blue	Sigma	Cat# B-8026
CheLuminate-HRP PicoDetect	Applichem	Cat# A3417,1200
cOmplete Tablets mini EDTA-free	Roche	Cat# 4693159001
DAPI - (4’,6-diamidino-2-phenylindol)	Sigma–Aldrich	Cat# D9542
DMEM (1×) + GlutaMAX	Gibco	Cat# 61965-026
Opti-MEM + GlutaMAX	Gibco	Cat# 51985-034
DMSO (Dimethylsulfoxide)	Roth	Cat# 4720
Doxycycline	Sigma–Aldrich	Cat# D9891-1G
ECL Prime	GE Healthcare	Cat# RPN2232
Entrectinib (RXDX-101)	Selleckchem	Cat# S7998
Fetal calf serum (FCS)	Linaris	Cat# AB-6000
Gene Ruler - 1 kb DNA ladder	Fermentas	Cat# SM0311
Gene Ruler - 100 bp DNA ladder	Thermo Scientific	Cat# SM0241
Hank’s Balanced Salt Solution (HBSS)	Gibco	Cat# 14170
HD Green Plus DNA stain	Intas	Cat# ISII-HDGreen Plus
HEPES	Sigma	Cat# H4034-25G
Immobilon Western HRP substrate	Merck Milipore	Cat# P90720
Immun-Blot PVDF Membrane	BioRad	Cat# 1620177
K252a	Abcam	Cat# ab120419
Larotrectinib (LOXO-101) sulfate	Selleckchem	Cat# S7960
Lipofectamine 2000	Invitrogen	Cat# 11668-019
Luminaris HiGreen qPCR Master Mix	Thermo Fisher Scientific	Cat# K0992
4–15% Mini-PROTEAN® TGX™ Precast Protein Gels, 10-well, 30 µl	BioRad	Cat# 4561083 S
Nonidet P40 Substitute	Sigma	Cat# 74385
Paraformaldehyde	Merck	Cat# A113 13
Penicillin-Streptomycin (5000 U/mL)	Gibco	Cat# 15070-063
Puromycin	InvivoGen	Cat# ant-pr-1
Polyacrylamide 30%	BioRad	Cat# 1610158
Poly-D,L-ornithine hydrobromide (PORN)	Sigma	Cat# P8638
Poly-L Lysine hydrobromide (PLL)	Sigma	Cat# P2636
Prestained protein ladder	Thermo Scientific	Cat# 26616
SDS (Sodiumdodecylsulfate)	Applichem	Cat# A2572,1000
Superscript III RT	Invitrogen	Cat# 18080
TEMED (N,N,N′,N′-Tetramethylethylenediamine)	Merck	Cat# UN2372
Tris base	Roth	Cat# 4855.3
Tris HCl	Roth	Cat# 6771.1
Triton X100	Sigma–Aldrich	Cat# 9002-93-1
TrypLE-Express	Gibco	Cat# 12605-010
Tween 20	Sigma	Cat# P1379
Experimental models: Cell lines		
HEK293	ATCC	Cat# 305
U87MG	ATCC	Cat# HTB-14
NIH-3T3	Cell Lines Services	Cat# 400101
Critical commercial assays		
Pierce BCA Protein Assay Kit	Thermo Scientific	Cat# 23225
NucleoBond Xtra Midi EF	Macherey Nagel	Cat# 740420
Monarch-PCR & DNA Cleanup Kit	New England Biolabs	Cat# T1030S
Monarch-DNA Gel Extraction Kit	New England Biolabs	Cat# T1020S
RNeasy Mini Kit	Qiagen	Cat# 74104
Quick Blunting Kit	New England Biolabs	Cat# E1201
Quick Change II XL - Site directed mutagenesis Kit	Agilent Technologies	Cat# 200521
Agilent RNA 6000 Nano Kit	Agilent Technologies	Cat# 5067-1511
TruSeq Stranded mRNA Library Prep	Illumina	Cat# 20020595
Agilent DNA 1000 Kit	Agilent Technologies	Cat# 5067-1504
Software and algorithms		
ImageJ	https://imagej.nih.gov/ij/	RRID:SCR_003070
Adobe Photoshop CS5 Extended	http://www.adobe.com/	RRID:SCR_014199
OriginPro	https://www.originlab.com/	RRID:SCR_014212
GraphPad Prism	http://www.graphpad.com/	RRID:SCR_002798
Olympus Fluoview – FV10-ASW 3.0	http://www.olympus-lifescience.com/en/	RRID:SCR_014215
Leica LAS AF Image Acquisition Software	http://www.leica-microsystems.com/	RRID:SCR_013673
LightCycler Roche	http://www.roche-applied-science.com/	RRID:SCR_012155
Python Programming Language	https://www.python.org/	RRID:SCR_008394
FACS: Attune Nxt Nxt analysis software	https://www.thermofisher.com/us/en/home/life-science/cell-analysis/flow-cytometry/flow-cytometers/attune-acoustic-focusing-flow-cytometer.html	RRID:SCR_019590
Illumina NextSeq RTA 550 System (v3.7.17)	https://www.illumina.com/systems/sequencing-platforms/nextseq.html	RRID:SCR_016381
bcl2fastq2 - Illumina (v2.20.0.422)	https://support.illumina.com/sequencing/sequencing_software/bcl2fastq-conversion-software.html	RRID:SCR_015058
Cutadapt (v2.5)	(Martin 2011)	RRID:SCR_011841
STAR (v2.7.2b)	(Dobin et al., 2013)	RRID:SCR_004463
featureCounts (v1.6.4)	(Liao et al.,2014) http://bioinf.wehi.edu.au/featureCounts/	RRID:SCR_012919
DESeq2 (v1.24.0)	(Love et al., 2014) https://bioconductor.org/packages/release/bioc/html/DESeq2.html	RRID:SCR_015687
g:Profiler	http://biit.cs.ut.ee/gprofiler/	RRID:SCR_006809
Reactome	https://reactome.org/	RRID:SCR_003485

### Indirect immunofluorescence

Coverslips (10 mm, Marienfeld) were placed in 4-well tissue culture dishes (Greiner) and coated with 0.1 mg/ml poly-L-lysine (PLL, Sigma). Cells were seeded on coverslips (150,000 cells/dish). After transfection and indicated expression times, the cells were fixed with PBS buffered with 4% paraformaldehyde (pH 7.4) for 15 min at 37 °C. The blocking solution contained 1% bovine serum albumin (BSA) or 10% horse serum in PBS supplemented with 0.1% Triton X100 and 0.1% Tween 20. Antibodies were diluted in a blocking solution. The coverslips were washed eight times with 0.1% Tween 20/PBS). Fluorochrome-conjugated secondary antibodies (Alexa Fluor 488, Cy3, and Cy5 (Jackson Laboratories) were used for 1 h at room temperature (21–23 °C). Cell nuclei were labeled with DAPI (2 mg/ml stock solution, freshly diluted 1:5000 in PBS) for 5 min at RT. For some experiments, cells were further incubated for 30 min with Alexa-670-phalloidin (Cytoskeleton #PHDN1) to label the actin filaments. Following DAPI treatment, the cells were washed twice with PBS. The coverslips were mounted using Aquapolymount (Polysciences).

### Confocal laser scanning microscopy and image processing

Images were acquired using an inverted IX81 microscope equipped with an Olympus FV1000 confocal laser scanning system, FVD10 SPD spectral detector, and diode lasers of 405, 473, 559, and 635 nm. All images shown were acquired with an Olympus UAPO 20x (air, numerical aperture 0.70) or UPLSAPO 60x (oil, numerical aperture:1.35) objective. For high-resolution confocal scanning, a pinhole setting representing a single Airy disc was used. In the case of high-resolution imaging, confocal settings were chosen to achieve an optimum resolution of at least three pixels per feature in the x-y direction. In the z-direction, 300 nm steps were used. 12-bit z-stack images were processed by maximum intensity projection and adjusted for brightness and contrast using Image J software (Rasband, W.S., ImageJ, U.S. National Institutes of Health, Bethesda, Maryland, USA, https://imagej.nih.gov/ij/) [47]. The images are shown as RGB images (8-bit per color channel). Fluorescence images were processed for the final presentation using Adobe Photoshop CS5.

### Live cell imaging

For live cell imaging experiments, µ-high 35 mm Ibidi dishes (Ibidi # 81156) were utilized. These dishes were first coated with poly-L-ornithine (PORN; Sigma). HEK293 cells were grown on coated dishes at 100,000 cells per dish. Cells were transfected with TrkB and GFP-actin plasmids. After 24 h, the old medium was replaced with pre-warmed HEPES-buffered DMEM (containing 10% FCS, 100 units/ml penicillin, 100 µg/ml streptomycin, and 1 mM sodium pyruvate). Cells were imaged using a Leica SP5 inverted confocal microscope equipped with Leica objectives (HC PL Apo ×20/0.7; HCX Apo ×60/1.4–0.6 oil). GFP actin was excited using a 488 nm laser line. Fluorescence was detected using a spectral detector (12 bit) at Airy disc1 settings.

### TrkB kinase domain modeling

The PDB entry 4AT4 was chosen as the basis for the modeling process. MD runs were performed using the gromacs 5.1 package. After solvation, addition of ions, energy minimization, and equilibration, productive MD was run for 1 ns. TrkB variants were generated using the COOT program and simulated using the same protocol as the original model.

### Western blot analysis

HEK293 cells were grown in 35 mm cell culture dishes (Falcon). A total of 200,000 cells per dish were transfected with the DNA plasmids using Lipofectamine 2000. Cells were lysed at 30 h on ice using a cell scraper and 150 µL cold lysis buffer (1% NP40, 50 mM HEPES pH 7.5, 150 mM NaCl, 10% glycerol, 1 mM sodium fluoride, 10 mM sodium pyrophosphate, 2 mM sodium orthovanadate, 5 mM EDTA, supplemented with one EDTA-free protease inhibitor mini tablet/5 ml of buffer (Roche Cat#4693159001). For lysis of SQSTM1-NTRK2 fusion proteins, we also used RIPA buffer (1% NP40, 0.5% sodium desoxycholate, 0.1% SDS, 150 mM NaCl, 50 mM Tris, 5 mM EDTA, pH 7.4). The lysates were incubated on ice for 15 min, sonicated twice for 5 s (Hielscher Sonifier UP50, M1 sonotrode, 80% power, 10 cycles of 0.5 s), and placed back on ice for 10 min. The lysates were centrifuged for 5 min at 4 °C and 15,000 × *g*. The protein concentrations of the samples were determined using a Pierce BCA protein assay kit (Thermo Scientific). For SDS-PAGE, the supernatant was mixed with the Laemmli sample buffer and heated for 5 min at 95 °C. For separation of SQSTM1-NTRK2 monomers, 2x Laemmli sample buffer was used. After SDS-PAGE, the proteins were transferred onto PVDF membranes (Immun-Blot, Bio-Rad). Western blotting was performed at 4 °C using Mini Trans-Blot Cell Assembly (BioRad) at 25 V (constant) for 15 h or 100 V (constant) for 90 min. The blocking solution contained 5% milk powder (BioRad) in Tris-buffered saline containing 0.2% Tween 20. After blocking for 30 min at RT, the primary antibody was used in the blocking solution for 3 h at RT. The blots were washed three times (20 min each) with TBST and then incubated with a secondary antibody for 2 h at RT. Blots were developed using ECL (Immobilon Western HRP Substrate, Merck Millipore) and X-ray films (Fujifilm Super RX). For quantification, the developed X-ray films were first photographed with a 12 MP Canon camera by placing them on a white transilluminator plate inside the PeqLab Gel Documentation system and were saved as 12-bit images. The integrated densities were then calculated for the protein bands using ImageJ software. For some experiments, Western visualization was performed with a Chemidoc Touch imaging system (BioRad).

U87MG cells expressing TrkB constructs were seeded at a density of 20,000 cells per well on 35 mm cell culture dishes (Falcon). One day after seeding, 1 mg/ml doxycycline was added to induce the expression of the corresponding TrkB-related constructs (Dox on). DMSO was used as the control. 24 h after Dox induction, cells were lysed in the same way as described above. In this case, 4–15% Gradient Gels (BioRad Cat# 4561083 S) were used for SDS-PAGE.

For the analysis of human glioblastoma tissue, frozen tissue samples were briefly thawed at RT and washed twice in 1x PBS. Small chunks were then distributed into microfuge tubes and lysed with cold lysis buffer, as described above. The sonication and centrifugation steps were repeated until a homogenous suspension was obtained. Samples were loaded onto SDS-PAGE gels after BCA protein quantification and then immunoblotted as described above. We investigated 20 µg of protein per lane (cell lines) or 40 µg/lane (glioblastoma or brain tissue).

### Patient glioblastoma tissue

The retrospective investigation of biomarkers in glioblastoma tissue samples using immunohistochemistry and molecular biology methods was approved by our institutional Ethics Committee (#103/14). Glioblastoma tissues were harvested during the brain surgery. Fresh tissue was snap-frozen in tissue tek (O.C.T compound, Sakura) at −30 °C. For fast histological examination, cryosections were stained with hematoxylin and eosin. After histological examination, the tissue-tek embedded, tumor-positive tissue was stored at −80 °C in a local tissue bank (Institute of Pathology, Department of Neuropathology, University Hospital, Würzburg). Tissue categorized as glioblastoma, IDH-wildtype, or CNS WHO grade 4 was selected and used for Western blotting, qPCR, or immunohistochemistry. According to the new WHO classification [28], one sample was categorized as astrocytoma, IDH-mutant, CNS, or WHO grade 4. For immunofluorescence labeling, the tissues were embedded in paraffin according to standard procedures. Paraffin-embedded frontal brain postmortem tissue was used as the control.

### Immunohistochemistry (glioblastoma tissue)

For immunohistochemical staining of glioblastoma tissues, formalin-fixed paraffin-embedded sections were deparaffinized in 100% xylene and rehydrated using a graded alcohol series (100%, 96%, and 70% for 5 min each). For antigen retrieval, the specimens were heat-treated for 10 min in 20 mM citric acid buffer (pH 6.0) in a pressure cooker. The sections were rinsed with dH_2_0 and 1× TBS and blocked with a solution containing 10% horse serum and 0.3% Triton X100 in TBS, for 1 h at RT. The tissues were then incubated overnight at 4 °C with primary antibodies. For glioblastoma IHC, the following antibodies were used: anti-human Nestin (R&D Systems, Cat# MAB1259, 1:2400), goat anti-TrkB (R&D Systems, Cat# AF1494, 1:1000), rabbit anti-pY674/675-TrkA (anti-pY706/707–TrkB) C50F3 (1:500). All antibodies were diluted in antibody dilution buffer (DCS - Innovative Diagnostik System, Hamburg, Germany). The next day, the sections were washed twice in TBS and treated with the corresponding secondary antibodies (Table 2) for 3 h at RT. The sections were washed twice in 1× TBS and stained with DAPI (2 mg/ml stock solution, freshly diluted 1:5000 in 1× TBS) for 5 min. The sections were washed twice in 1× TBS and embedded in Aquapolymount (Polysciences). Kidney tissue was used as a positive control for the primary antibody against Nestin. Biopsy samples from healthy donors (autopsies) were used as negative controls. Cross-reactivity of secondary antibodies was tested by using secondary antibodies in the absence of the corresponding primary antibodies.

### RNA isolation and quantitative RT–PCR

Total RNA from U87MG cells was prepared using an RNeasy Mini Kit (Qiagen). For glioblastoma, frozen samples were thawed and washed twice in 1x PBS. Small chunks were then distributed into microfuge tubes and RNA isolation was performed using the RNeasy Mini Kit (Qiagen). To generate cDNA, a Superscript III Reverse Transcriptase First Strand Synthesis Kit (Cat# 12371-019) was used with 500 ng RNA and 50 ng random hexamer primers. The cDNA reaction was 5-times diluted in 10 mM Tris-HCl, pH 8.5 containing 1 mg/ml BSA. Light cycler 96 Detection System (Roche) was used to perform RT‐qPCR using the Luminaris HiGreen qPCR Master Mix Kit (Thermo Fischer) was used with a standard amplification protocol (denaturation:95 °C, 15 s; annealing:60 °C, 30 s; 72 °C, 30 s) and an equivalent of 5 ng RNA as input. The primer information is provided in Table 3. The signals were normalized to RNA Pol II expression levels using the ΔΔCT method.

**Table 3:** Primer sequences.

Primer name	Reference sequence	Amplicon (in reference)	Primer sequence
hTrkB splice^a^	NM_006180	(position 1836–2019)	5´– CTGTGGTGGTGATTGCGTCT –3´ 3´– GGGCTGGCAGAGTCATCATC –5´
RNApol II (POLR2A)	NM_000937	(position 4466–4732)	5´– GCACCACGTCCAATGACAT –3´ 3´– GTGCGGCTGCTTCCATAA –5´

^a^The primers span two splice sites in the human NTRK2 gene. This includes the splice site for TrkB-kinase versus truncated TrkB-T1.

### RNA sequencing

RNA quality was checked using a 2100 Bioanalyzer with an RNA 6000 Nano Kit (Agilent Technologies). The RIN for all the samples was ≥9.0. DNA libraries suitable for sequencing were prepared from 500 ng of total RNA with oligo-dT capture beads for poly-A-mRNA enrichment using the TruSeq Stranded mRNA Library Preparation Kit (Illumina), according to the manufacturer’s instructions (½ volume). After 14 cycles of PCR amplification, the size distribution of the barcoded DNA libraries was estimated to be ~300 bp using electrophoresis on Agilent DNA 1000 Bioanalyzer microfluidic chips.

Sequencing of pooled libraries spiked with 2% PhiX control library was performed at 27–45 million reads/sample in single-end mode with 100 nt read length on the NextSeq 2000 platform (Illumina) using a P3 sequencing kit. Demultiplexed FASTQ files were generated using the bcl2fastq2 v2.20.0.422 (Illumina). To ensure high sequence quality, Illumina reads were quality- and adapter-trimmed using Cutadapt [48] version 2.5 using a cutoff Phred score of 20 in the NextSeq mode, and reads without any remaining bases were discarded (command line parameters: --nextseq-trim = 20 -m 1 -a AGATCGGAAGAGCACACGTCTGAACTCCAGTCAC). Processed reads were subsequently mapped to the human genome (GRCh38.p13 primary assembly and mitochondrion) using STAR v2.7.2b with default parameters based on the RefSeq annotation version 109.20210226 for GRCh38.p13 [49]. Read counts at the exon level summarized for each gene were generated using featureCounts v1.6.4, from the Subread package [50]. Multi-mapping and multi-overlapping reads were counted as strand-specific and reversely stranded with a fractional count for each alignment and overlapping feature (command line parameters: -s 2 -t exon -M -O --fraction). The count output was used to identify differentially expressed genes using DESeq2 [51] version 1.24.0. Read counts were normalized using DESeq2, and fold-change shrinkage was applied by setting the parameter “betaPrior = TRUE”. Differential gene expression was assumed at an adjusted *p*-value (padj) after Benjamini-Hochberg correction < 0.05, and |log2FoldChange | ≥ 1. For gene set enrichment analysis based on lists of significant genes, we considered the DESeq2 log2FoldChange of all analyzed genes. g:Profiler [52] was used to perform functional enrichment analyses based on Reactome biological pathways [53]. The significance threshold was set using the Benjamini-Hochberg FDR with a user threshold of 0.05.

### Statistical analysis

Statistical analyses were performed using Origin Pro 2019b. The data are presented as the standard error of the mean ( ± SEM). Column statistics were run to check for a Gaussian distribution to decide whether to use parametric or non-parametric tests. If one of the groups failed the normality test or if the value number was too small to run the normality test, non-parametric tests were chosen for further analysis. Normality was tested using the Shapiro-Wilk test and equality of variances (Levene’s test), and based on the results either the 2-sample *t*-test or the Mann–Whitney *U*-test was used. The results were considered statistically significant at *p* < 0.05.

## Results

### TrkB mutants and antibody specificity testing

**Fig. 1:**
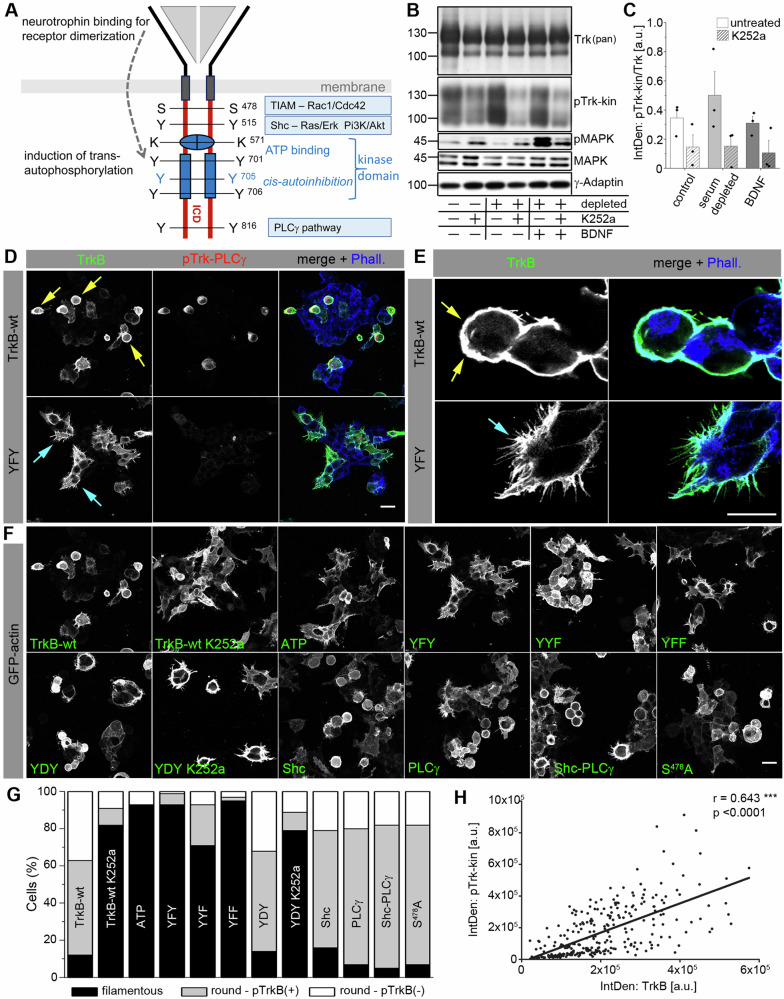
Abundance-dependent activation of TrkB kinase and changes in actin morphology strongly depend on phosphorylation in the YxxxYY-motif. **A** Model depicting TrkB kinase signaling. Neurotrophin (BDNF/NT-4)) binding induces conformational changes and supports receptor dimerization. The kinase is released from cis-autoinhibition, ATP binds to the intracellular kinase domain and allows trans-autophosphorylation. Three tyrosine residues in the consensus motif YxxxYY, Y^701^, Y^705^, and Y^706^ are phosphorylated in the activation loop of the receptor. ATP-binding and phosphorylation of the YxxxYY motif occur upstream of autophosphorylation and downstream signaling. In the intracellular domain (ICD), phosphorylation of Y^515^ recruits Shc and activates Ras/ERK and Pi3K-Akt pathways. Y^816^ forms an adapter site for PLCγ. S^478^ signals to the TIAM-Rac1 pathway. **B** TrkB phosphorylation in the absence of BDNF is unaffected by serum depletion. Western blotting of whole-cell lysates generated from HEK293 cells expressing TrkB. Control cultures were maintained in serum before total lysates were produced. The cell cultures were treated as indicated. Serum depletion was performed for 3 h. To inhibit TrkB kinase activity, the cultures were preincubated with 150 nM K252a, a Trk kinase inhibitor, for 30 min. DMSO was used as the solvent control. When indicated, cells were also treated with 20 ng/ml BDNF for 15 min and compared with BDNF-stimulated cells under K252a treatment. **C** Quantification of Western blots for pTrkB-kin normalized to the total Trk-kinase levels by densitometry. The relative integrated densities are also presented. K252a treatment for 30 min caused a reduction in TrkB phosphorylation levels under control, serum-depleted, and BDNF stimulation conditions. Bar graph: Mean ± SEM, overlaid with single data points; *n* = 3. **D** In the absence of neurotrophins, TrkB overexpression induces TrkB phosphorylation and changes cell morphology. Immunofluorescence of the TrkB receptor (green) and pTrk-PLCγ (red). F-actin was labeled with Acti-stain-670 phalloidin (blue). HEK293 cells were transfected with either TrkB- or TrkB-YFY kinase mutant. The cells were immunostained for 30 h. Yellow arrows indicate round pTrk-positive cells. Cyan arrows indicate filamentous pTrk-negative cells. Confocal images; scale bar:25 µm. **E** Filopodia phenotype of TrkB-expressing cells (high-resolution confocal stack image). **F** Round-shaped cells expressing kinase-active TrkB mutants. Immunostaining of HEK293 cells expressing either TrkB-wt or TrkB mutants (for details see Fig. S1). Cells expressing TrkB-wt or the constitutively active mutant TrkB-YDY were also treated with K252a. Typically, filamentous cells express kinase-dead ATP mutants of TrkB or YxxxYY mutants (YFY and YFF). Treatment with 150 nM K252a for 30 min reversed the round shape of the cells expressing TrkB-wt or TrkB-YDY. Confocal images; scale bar: 25 µm. **G** Quantification of the percentage of cells showing either a round shape or typical filopodia. Round cells were further subdivided into those that were either positive (+) or negative (−) for pTrk. Data were acquired from ten fields of view in three biological replicates (*n* = 3). Scale bar: 25 µm. **H** TrkB phosphorylation is correlated with TrkB expression. Linear positive correlation of the integrated density of anti-TrkB and anti-pTrkB-kin immunoreactivity in TrkB-expressing HEK293 cells. Immunolabels per cell were measured as the integrated density per cell from the maximum intensity projection images of confocal z-stacks. Single-cell data, *n* = 280 cells; data collected from 20 confocal image fields and 4 cell cultures (*n* = 4).

First, we performed experiments using cell lines. To characterize the functional prerequisite of TrkB, we used site-directed mutagenesis to clone a series of mouse TrkB mutants (based on the reference sequence: NP001020245, Fig. S1). Figure 1A shows a model of the TrkB receptor, highlighting the critical structural components, phosphorylation sites, and binding sites of our antibodies against TrkB. The specificity and properties of the antibodies were investigated with the help of TrkB mutants and other Trk family members using immunofluorescence (Fig. S2), and western blotting. These experiments showed that all anti-phospho Trk (pTrk)-specific antibodies could detect phospho-active TrkB. However, specific properties of the antibodies need to be considered when used in western blotting or immunocytochemical labeling experiments, or when used on TrkA or TrkC, or certain TrkB mutants. The results of the specificity tests are summarized in Table S1. In our study, we refer to the tyrosine triplet of TrkB as follows: YYY = Y^701^, Y^705^, and Y^706^ (Fig. 1A).

### TrkB kinase activity causes abundance-dependent changes in cell morphology

We transiently transfected HEK293 cells with wild-type TrkB (TrkB-wt) and performed western blotting. As expected, TrkB was highly expressed and constitutively active (top row in Fig. 1B). Immunoblotting of pTrk was performed using anti-pTrk-kin, an antibody specific for phosphorylation at Y^705^ in TrkB (Table S1). Total and pTrkB appeared at 130 kDa, representing the mature glycosylated TrkB kinase, and 90 kDa, representing a typical immature underglycosylated TrkB. To exclude the possibility that serum components in the growth medium might transactivate TrkB in the absence of neurotrophins, we performed a 3 h serum-depletion. After 3 h of serum starvation, the cells started to detach, and MAPK phosphorylation downstream of TrkB was reduced (Fig. 1B). However, serum depletion did not reduce TrkB phosphorylation at Y^705^ (Fig. 1B, C). HEK293-TrkB cells were then treated for 30 min with 150 nM K252a, a prototypical Trk inhibitor [54]. K252a acutely reduced TrkB phosphorylation both under control conditions and after 3 h of serum-depletion (Fig. 1B, C). Stimulation with 20 ng/ml BDNF was unable to increase pTrkB-kin levels after serum depletion or in the presence of K252a (Fig. 1B, C). Although serum depletion was insufficient to halt the constitutive phosphorylation of TrkB, it resulted in reduced MAPK phosphorylation downstream of TrkB (Fig. 1B). ANA-12, a modern TrkB antagonist [55] targeting the receptor domain of TrkB, could not block the ongoing TrkB kinase activity (Fig. S3). Hence, we stuck with Trk kinase inhibitors for the following experiments.

In the next set of experiments, after an expression time of 30–48 h, the transfected cells were triple-labeled for TrkB, pTrkB-PLCγ and filamentous actin (F-actin) and observed under a confocal microscope. F-actin was labeled with a phalloidin-Cy5 conjugate, which helped stain the gross morphology of the cells. Most TrkB-wt-expressing cells had a round cell body and showed a pronounced pTrk signal (Fig. 1D). In contrast, the Y^705^-TrkB mutant (TrkB-YFY) did not show obvious phosphorylation at the PLCγ-site (pPLCγ), indicating a reduction in autophosphorylation (Fig. 1D) and neither the round cell shape. Instead, TrkB-YFY-expressing cells formed filopodia (Fig. 1E). This indicates that Y^705^ is important for regulating cytoskeletal features of constitutively active TrkB.

Next, we determined the potency of diverse TrkB mutants in affecting F-actin in HEK293 cells (Fig. 1F, G). Transfection of cells with the kinase-dead TrkB mutant (TrkB-ATP) or mutants of kinase Y^705^ (TrkB-YFY and TrkB-YFF) did not alter actin/TrkB+ filopodia formation and the cells showed, like untransfected HEK293 cells, typical filopodia. However, cells expressing either TrkB-wt or any of the other TrkB mutants tested for the Shc, PLCγ or TIAM/Rac1/CDC42 interaction sites showed a roundish cell shape (Fig. S4). In TrkB-wt cells and in cells expressing phosphomimic TrkB (TrkB-YDY), the roundish cell phenotype could be reversed by K252a within 30 min. This shows that ongoing kinase activity is involved in the signaling process. For downstream signaling of TrkB, Shc- and PLCγ-adapter sites are crucial [7, 8]. However, the double mutant TrkB-Shc-PLCγ disrupted the filopodia-like phenotype (Figs. 1F, G, S4). Thus, we can say that the classical Shc/PLCγ signaling pathway of TrkB did not cause the alterations in cell morphology.

To test the effect of receptor abundance on phospho-kinase activity, we overexpressed TrkB in HEK293 cells and labeled them with either anti-TrkB or anti-pTrk-kin. We determined the integrated density of the corresponding immunolabels at the single-cell level using confocal microscopy. The data confirmed a linear, statistically significant correlation between TrkB abundance and pTrk-kin intensity (Fig. 1H).

It has been long known that the TrkB protein is expressed as either the TrkB receptor kinase or the kinase-deficient TrkB splice isoform TrkB-T1 [56, 57]. TrkB-T1 consists of complete extracellular and transmembrane domains but carries only a short cytoplasmic tail of 23 amino acids. Overexpression of TrkB-T1 [58] did not cause round cell morphology and did not destroy filopodia formation (Fig. S5). Overexpression of other Trk kinase family members, TrkA and TrkC, resulted in a roundish cell shape (Fig. S5). TrkB-wt overexpression led to the constitutive activation of both immature TrkB (90 kDa) and mature TrkB (130 kDa) (Fig. S5). Mutating the 12 predicted N-glycosylation sites in the receptor domain did not block constitutive TrkB activation (Fig. S5). Finally, TrkB cell surface labeling confirmed the presence of phospho-active TrkB at intracellular sites in the absence of a ligand (Fig. S5). This shows that glycosylation of the extracellular domain and trafficking effects caused by glycosylation does not prevent constitutive TrkB activation.

As the pTrk-kin antibody did not detect overexpressed TrkC, we also tested other anti-pTrk antibodies on TrkA and TrkC (Fig. S6). This confirmed that all NTRK family members became constitutively active by overexpression and caused a roundish cell phenotype, as long as they have intact kinase domains.

### Actin filopodia formation is disturbed in TrkB overexpressing cells

**Fig. 2:**
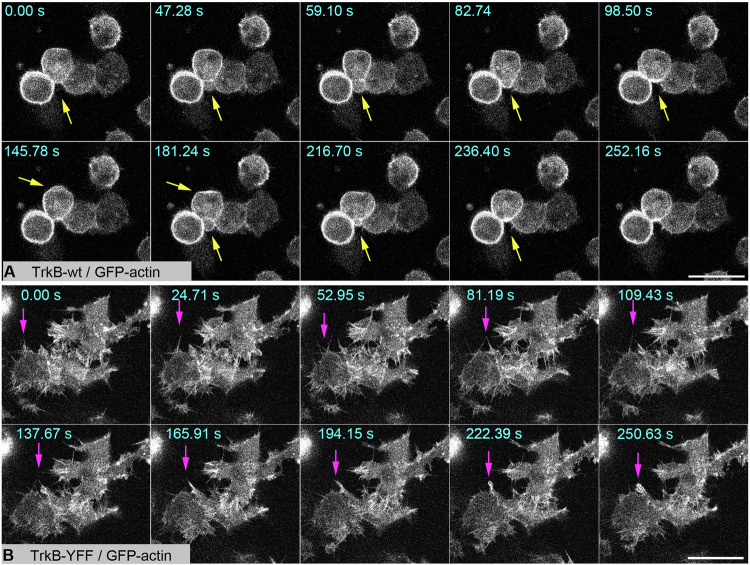
Mutating of the YxxxYY-motif in TrkB restores actin filopodia dynamics. **A**, **B** Overexpression of TrkB kinase induces round-shaped cell morphology and loss of actin filopodia dynamics. HEK293 cells co-expressing GFP-actin and TrkB-wt (in **A**) or TrkB-YFF mutants (in **B**). Time-lapse images (in seconds) are shown. Living cells were imaged using a confocal laser-scanning microscope. GFP-labeled-actin was excited with a 488 nm laser, and fluorescence was detected using a spectral detector (510–570 nm). **A** Arrows point to a roundish cell that forms typical blebs. **B** The arrow indicates typical dynamic filopodia labeled by GFP-actin. Videos can be found in the supplement. Scale bar: 25 µm.

To better visualize the actin phenotype of TrkB-expressing cells, we performed confocal live-cell imaging (Fig. 2). Cells were co-transfected with TrkB, TrkB mutants (representatively shown here is the mutant TrkB-YFF), and GFP-actin. In TrkB-wt cells, two phenotypes were observed: (1) roundish cells exhibiting low GFP-actin dynamics and (2) cells forming bleb-like structures (Fig. 2A, Videos 1 and 2). Bleb formation is a type of cell motility observed when the cytoskeleton is decoupled from the plasma membrane [59]. In contrast, cells expressing TrkB-YFF showed typical actin filopodia dynamics (Fig. 2B, Videos 3 and 4).

### TrkB overexpression induces phosphorylation of focal adhesion kinase (FAK)

**Fig. 3:**
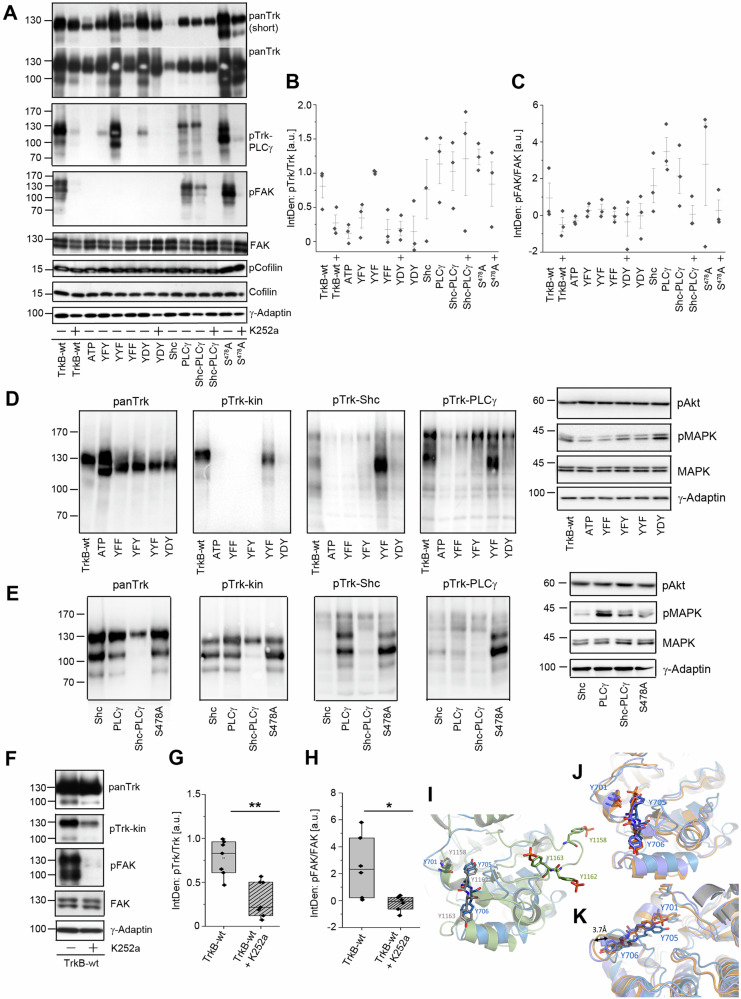
In HEK293 cells, constitutive active TrkB is upstream of focal adhesion kinase (FAK) phosphorylation. **A** Self-active TrkB-wt induces the phosphorylation of Focal Adhesion Kinase (FAK). Western blotting of whole-cell lysates generated from HEK293 cells expressing the indicated TrkB mutants. 150 nM K252a for 30 min was used to inhibit the Trk kinase activity. DMSO was used as the solvent control. Antibodies against total FAK, Cofilin and γ-Adaptin were used as loading controls. **B** Densitometric analysis of Western blots for pTrkB-kin normalized to the total TrkB levels in TrkB mutants. Means ± SEM, overlaid with single data points; *n* = 3. Note the high variability between biological replicates and failure of TrkB-kin activation in single samples. **C** pFAK signals were normalized to total FAK levels. The relative integrated densities are also presented. Means ± SEM, overlaid with single data points; *n* = 3. **D** Anti-pTrk signals in TrkB with mutations in the TrkB kinase domain or the ATP-binding site. **E** Anti-pTrk signals in TrkB with mutations in the Shc, PLCγ, or S478-adapter sites. **F** Self-active TrkB induces FAK phosphorylation This can be blocked by the Trk kinase inhibitor, K252a. Western blot of whole-cell lysates generated from HEK293 cells expressing TrkB-wt. **G** Quantification of Western blots for pTrkB-kin normalized to the total TrkB levels by densitometry. The relative integrated densities are also presented. K252a treatment reduces TrkB phosphorylation levels. Bar graph: mean ± SEM, overlaid with single data points; *n* = 7; 2-sample-*t*-test was performed: *t*(12) = 4.295, *p* = 0.00104. **H** Quantification of Western blots for pFAK normalized to the total FAK levels by densitometry. The relative integrated densities are also presented. K252a treatment reduced FAK phosphorylation levels. Bar graph: mean ± SEM, overlaid with single data points; *n* = 6; Mann–Whitney *U* test was performed: U = 32, *p* = 0.03064. **I**–**K** Modeling of TrkB. Superimposition (in I) of the TrkB MD model (blue) with the activated form of the insulin receptor (1gag, green). The YxxxYY motif is depicted in ball-and-stick mode. The YxxxYY motif of the autoinhibited insulin receptor is shown in gray and ball and stick modes. The backbone is omitted for clarity. The upper panel (in **J**) shows the superposition of the three MD models for TrkB. The wild-type is shown in blue. Phospho Y^705^ in orange and Y^705^F in purple. The YxxxYY motif is shown in the ball-and-stick mode, and the movement is indicated by an arrow (in **K**). UT.

The blebs observed in live actin cell imaging led us to ask whether self-active TrkB induces the phosphorylation of proteins involved in actin dynamics [60, 61]. Thus, we next overexpressed diverse TrkB mutants and probed the total cellular proteins with anti-Trk and anti-pTrk (PLCγ) (Fig. 3A, B). Furthermore, we tested for phosphorylated cofilin, a protein involved in F-actin reorganization, and focal adhesion kinase (FAK), a cytosolic tyrosine kinase that regulates focal adhesion site assembly, membrane protrusion formation, and cell motility [61]. We performed the experiment with the mutants three times, as the expression level of self-active TrkB is dependent on the expression levels (see Fig. 1) and can vary between biological replicates. Of note, the single TrkB-shc mutant was not sufficiently highly expressed in one experiment (Fig. 3A) but was pFAK-positive in the case of experimental repetition (Fig. 3B, C). Anti-phospho-cofilin immunoblotting was inconspicuous, but strong phosphorylation of FAK at Y^576/577^ was observed in TrkB-wt expressing cells (Fig. 3A, C). We also probed TrkB mutants with pTrk-specific antibodies (Fig. 3D, E). These experiments confirmed: (1) high specificity of anti-Trk-kin for kinase-active TrkB (2) phosphorylation of the Shc-PLCγ, YYF, and S478A mutants. FAK phosphorylation was inhibited by the Trk inhibitor, K252a (Fig. 3F–H).

Cells expressing the kinase-dead TrkB-ATP mutant or cells expressing TrkB with a site-directed missense mutation in Y^705^ or Y^706^ did not show FAK phosphorylation. Moreover, constitutively active TrkB-YDY, a protein typically associated with a round-cell phenotype, did not show pFAK activation. This indicates that the round cell phenotype depends on kinase-active TrkB, which can be mimicked by substituting Y^705^ with either D^705^ or E^705^. Notably, this did not necessarily lead to pFAK activation. The data showed that pFAK activation by TrkB in HEK293 cells depends on Y^705^ and Y^706^. We attempted to co-immunoprecipitate TrkB and pFAK, but the results were negative.

### Structural features of TrkB with phosphorylated or unphosphorylated Y705

We had a closer look at Y^705^ utilizing a TrkB crystal structure (pdb code 4AT4) and asked whether there are unusual structural features in TrkB in comparison to TrkB-YFY or phosphorylated Y^705^. We used the TrkB model for molecular dynamics (MD, GROMACS) and performed a 1 ns run. The resulting structure was then compared with the kinase domain of the insulin receptor (1 gag), which provided a reference for the fully triple phosphorylated autoinhibition loop (Fig. 3I). We observed large structural rearrangements between the TrkB model (blue) and activated insulin receptor (green). The inactive form of the insulin receptor closely resembled the conformation of the MD TrkB model, suggesting a similar autoinhibition state (Fig. 3I). To investigate this further, we conducted MD runs using two TrkB variants (phospho Y^705^ and Y^705^F) and compared all three TrkB-related models. In silico phosphorylation of Y^705^ in the TrkB model (orange) suggested that phosphorylation alters the position of the autoinhibition loop by shifting the YxxxYY motif (3.7 Å at Gly^712^ located at the tip of the loop; Fig. 3J, K). A similar change in loop position was observed for the Y705F variant (purple). These small but significant differences indicate structural transitions in the receptor structure that may underlie TrkB activation via ligand-independent release from cis-autoinhibition upon overexpression.

### The intracellular domain of TrkB is sufficient to induce FAK phosphorylation

**Fig. 4:**
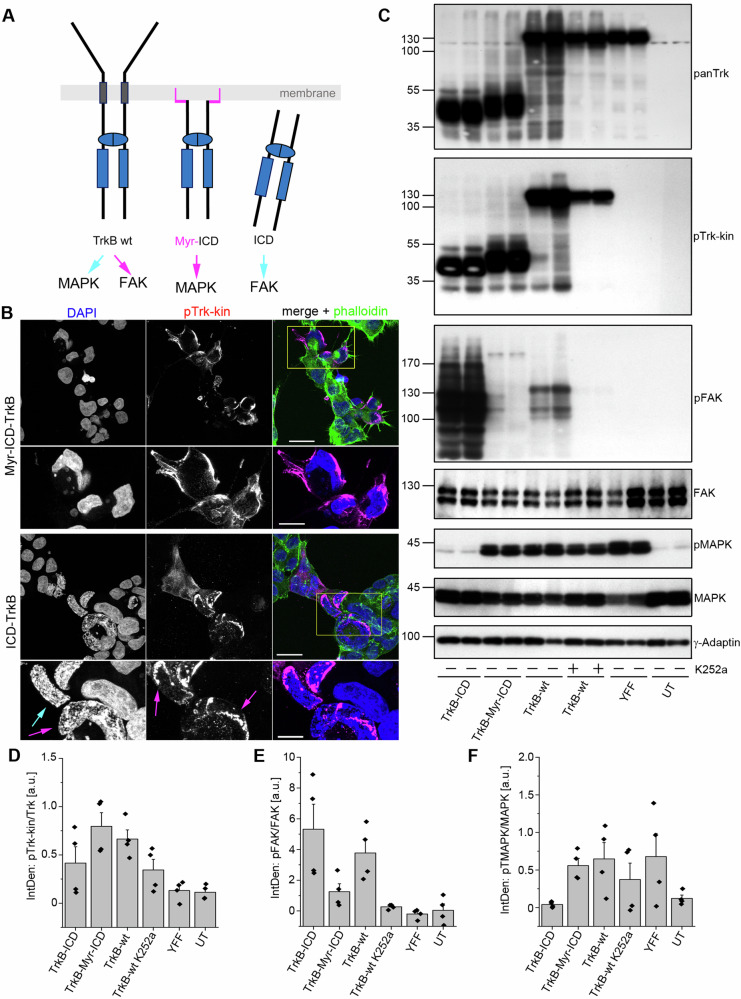
The intracellular domain (ICD) of TrkB transduces signals to FAK, while a membrane-targeted ICD of TrkB is linked to MAPK phosphorylation. **A** Model depicting intracellular kinase-active domain constructs of TrkB. In Myr-ICD, an N-terminal myristoylation motif with a glycine-serine (GGSGG)-linker was used for plasma membrane targeting. The ICD (intracellular domain) construct lacks ligand-binding and transmembrane domains. The model includes the results of the experiment. Constitutively active TrkB-wt signals to MAPK and FAK, whereas Myr-ICD activates MAPK, but not FAK. ICD activates FAK but not MAPK. **B** TrkB-ICD and TrkB-Myr-ICD undergo self-activation but differ in their cellular localization patterns. Immunofluorescence of pTrk-kin (magenta), DAPI (blue) and Acti-stain-670 phalloidin (phall, green). HEK293 cells were immunostained 30 h after transfection. TrkB-ICD is preferentially observed at intracellular sites. The Myr-ICD construct showed typical plasma membrane targeting as expected. Note morphological changes in DAPI staining of ICD-expressing cells, indicating changes in chromatin compaction (cyan arrows) and pTrk-kin clusters (magenta arrows). Scale bar: 20 µm. **C** TrkB-ICD, but not TrkB-Myr-ICD, induced FAK phosphorylation. Western blotting of whole-cell lysates generated from HEK293 cells expressing TrkB-ICD, TrkB-Myr-ICD, TrkB-wt, or TrkB-YFF. K252a was used to inhibit the Trk kinase activity. TrkB-wt expression increased MAPK and FAK phosphorylation. ICD signaled to FAK, but not to MAPK. Myr-ICD induced MAPK phosphorylation but failed to activate FAK. **D**–**F** Quantification of Western blots for pTrkB-kin normalized to total TrkB levels (in **D**), pFAK to total FAK (in **E**), and pMAPK to total MAPK (in **F**). The relative integrated densities are also presented. Bar graph: Mean ± SEM, overlaid with single data points; *n* = 4. UT untransfected control.

To further investigate the role of the TrkB intracellular domain in FAK phosphorylation, we generated two intracellular kinase domain (ICD) constructs. The TrkB-ICD construct carried the complete intracellular domain of TrkB (K^454^ to the C-terminal end) (Fig. 4A). Myr-ICD, consisted of the TrkB ICD coupled to an aminoterminal myristoylation (Myr)/S-acylation motif. This allowed targeting of Myr-ICD to the plasma membrane (Fig. 4A). We expressed both constructs in HEK293 cells, performed immunolocalization experiments (Fig. 4B). Immunolabelling confirmed that both TrkB-ICD and Myr-ICD were phosphorylated at Y^705^/Y^706^, although their cellular localization profiles differed (Fig. 4B). TrkB-ICD appeared at intracellular sites throughout the cytosol (Fig. 4B, magenta arrows), whereas Myr-ICD outlined the cell surface as expected (Fig. 4B). ICD-protein and Myr-ICD were found to be phospho-active and migrated at predicted relative molecular weight of 40–45 kDa (Fig. 4C). Surprisingly, the ICD domain was sufficient to cause dramatic induction of pFAK phosphorylation at Y^576/577^, but it did not induce MAPK phosphorylation (Fig. 4C–F). In contrast, Myr-ICD caused almost no FAK phosphorylation, but led to the activation of MAPK (Fig. 4C–F). TrkB-wt induced both pFAK and pMAPK, and K252a treatment caused a reduction of both pTrk and pFAK signals (Fig. 4C–F). The kinase mutant TrkB-YFF did not show pFAK activation but induced MAPK phosphorylation (Fig. 4C–F). From these data, we assumed that Myr-ICD, when targeted to the plasma membrane, can provide a platform for adapter proteins, thereby supporting a certain level of constitutive Ras/MAPK signaling, even in the absence of neurotrophins.

### A cytosolic *NTRK* fusion protein behaves like constitutive active TrkB

Some cancer-related *NTRK* fusion proteins lack the amino-terminal Trk receptor domain and are fused to cytosolic proteins [1, 37]. Structural features suggest that these proteins are localized at intracellular sites and become oncogenic drivers due to kinase activation [12]. We investigated whether such a intracellular NTRK-fusion protein behaves like our TrkB-ICD construct and synthesized a cancer-related *SQSTM1-NTRK2* reading frame. This NTRK-fusion oncogene was of specific interest for our research question because it carries the TrkB kinase domain and an intact C-terminus, while lacking the receptor domain, the juxtamembrane region, and the Shc adapter site for the MAPK/ERK pathway. Furthermore, it is also known to be found in diverse tumors of the central nervous system, including gliomas [26, 27, 37]. Recently, in was confirmed that patients with *SQSTM1-NTRK2*-positive tumors show complete response to Trk-inhibitor therapy with Larotrectinhib [62]. In *SQSTM1-NTRK2*, exon 1-to-5 of sequestosome 1 (SQSTM1), a multifunctional signaling adapter involved in autophagy, are fused to exons 16–20 of *NTRK2* [12, 27, 37]. This creates an open reading frame and links the amino-terminal part of SQSTM1 with the intracellular, soluble kinase domain of human TrkB. The aminoterminal fusion part of SQSTM1 encodes the Phox and Bem1 (PB1) domain, which provides self-interaction surfaces for oligomerization or hetero-dimerization with other PB1 domains [63, 64]. The part also contains the ZZ-domain, which serves as a multiprotein and RNA interaction hub [65].

**Fig. 5:**
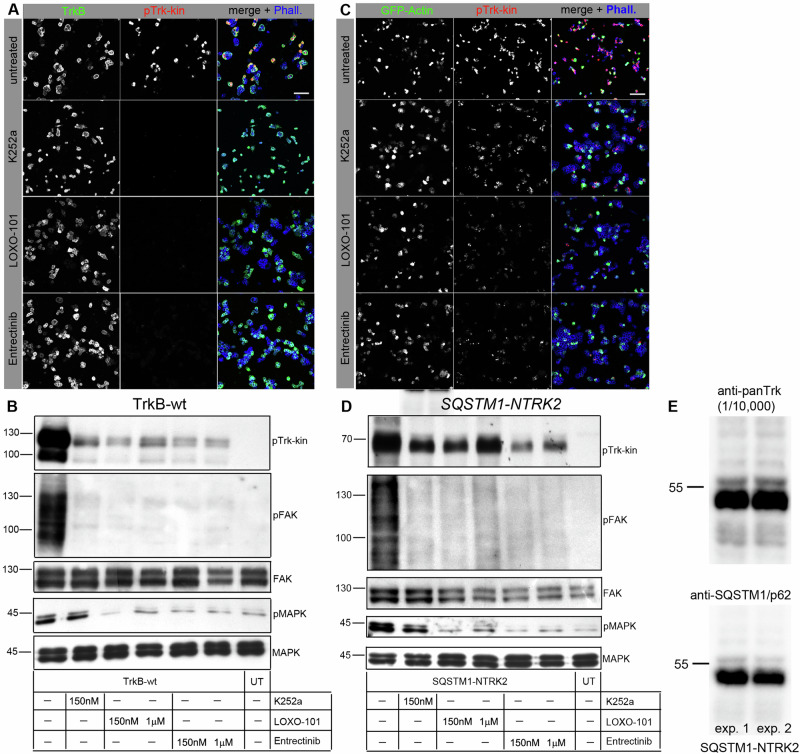
Treatment of TrkB-wt and SQSTM1-NTRK2 (a TrkB fusion product) with small molecule Trk inhibitors reduces or hinders downstream Trk activated pathways. **A**, **C** Immunostaining of HEK293 cells expressing TrkB-wt or *SQSTM1-NTRK2*. Immunofluorescence of TrkB (green), pTrk-kin (red) and Acti-stain-670 phalloidin (phall, blue). Cells were treated with small molecule Trk inhibitors like K252a, LOXO-101 and Entrectinib. TrkB self-activity is reduced upon inhibitor treatment. Confocal images; scale bar: 100 µm. **B**, **D** Western blotting of whole-cell lysates generated from HEK293 cells expressing TrkB-wt or *SQSTM1-NTRK2*. After transient transfection, the constructs were expressed for 30 h and then treated with small molecule Trk inhibitors like K252a, LOXO-101 and Entrectinib (concentrations are as depicted). Lysates were probed with the indicated antibodies via classical western blotting. Trk, FAK and MAPK activity was reduced upon inhibitor treatment. Downstream activity of FAK and MAPK was reduced upon inhibitor treatment. **E** Anti-SQSTM1/p62 detects the SQSTM1-NTRK2 fusion protein at the predicted relative molecular weight.

We expressed *SQSTM1-NTRK2* in HEK293 cells that showed constitutive phosphorylation. It caused a roundish cell phenotype, was able to induce phosphorylation of FAK^Y576/577^ and, in contrast to Trk-ICD, could also activate MAPK (Fig. 5). All these effects could be reversed with the Trk inhibitor K252a and the clinically approved Trk kinase inhibitors LOXO-101 (Larotrectinib) and Entrectinhib (Fig. 5). Thus, our data so far showed that both TrkB and the intracellular NTRK2-fusion oncogene (SQSTM1-NTRK2) could undergo constitutive activation and share atypical signaling patterns.

### Atypically active TrkB inhibits migration of U87MG cells

TrkB kinase is often seen as a promigratory receptor during cortex development or in brain tumors [16, 66]. However, we observed that constitutive TrkB signaling can also cause cell blebbing and can interrupt filopodia formation. Thus, we thought it made sense to next explore how self-active TrkB or intracellular NTRK fusion protein affect cell migration.

**Fig. 6:**
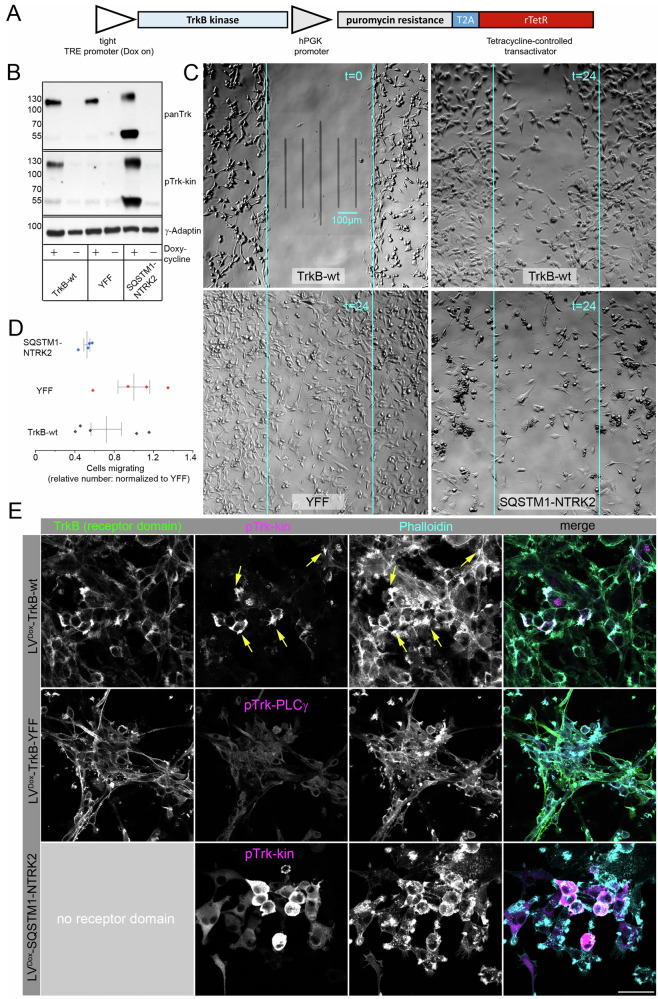
Constitutive active TrkB and the *SQSTM1-NTRK2* oncogene reduce migratory activity of U87MG cells. **A** Outline: inducible lentiviral expression vector. **B** Western blotting of whole-cell lysates generated from U87MG cells expressing the TrkB kinase construct. Cells transduced with the indicated lentiviral constructs were maintained as a polyclonal cell line, and puromycin was used to select transduced cells. In the absence of doxycycline, cells did not express the corresponding proteins. Doxycycline was added to induce Trk construct expression for 48 h. Constitutive activation of Trk was verified using pTrk-kin. γ−Adaptin was used as a loading control. **C** Representative phase-contrast microscopy images of the migration assay. U87MG cells stably expressing either TrkB-wt, TrkB-YFF, or the fusion protein SQSTM1-NTRK2 via the Tet-on system. The image in the upper left corner shows the cells in the culture after removing the silicone insert (*t* = 0). The other images represent the situation 24 h later. Note the morphological changes in TrkB-wt and SQSTM1-NTRK2 expressing cells versus TrkB-YFF. **D** Migratory activity of U87MG cells expressing the indicated Trk kinase constructs. The relative numbers of cells relative to the initial cell-free gap are shown. Cell counts were normalized to the mean number of TrkB-YFF-expressing cells. Migratory activity is shown relative to TrkB-YFF, which expresses the same structural protein domains as TrkB-wt but is mutated at Y^705^ and Y^706^ (see also S8); *n* = 4 biological replicates. **E** Immunostaining of U87MG cells expressing inducible TrkB-wt, TrkB-YFF, or the NTRK fusion construct *SQSTM1-NTRK2*. Immunofluorescence of the TrkB receptor domain (green), pTrk-kin (magenta), and Acti-stain-670 phalloidin (phall, cyan). Yellow arrows indicate constitutive pTrk close to F-actin. TrkB-YFF-expressing cells were labeled with anti-pTrk-PLCγ because the antibody-binding site of anti-pTrk-kin was mutated in this construct. *SQSTM1-NTRK2* does not have a receptor domain, as indicated. Confocal z-stack images; scale bar: 50 µm.

For this, we used human U87MG glioblastoma-like cells, which are commonly used in brain cancer research. U87MG (ATCC) cells are of CNS origin and carry bona fide glioblastoma-like characteristics [43]. The cells are migratory and are often used to determine how candidate proteins interfere with non-directed cell migration [67]. To better control protein abundance in this cell model, we expressed TrkB-wt, TrkB-YFY, and *SQSTM1-NTRK2* using a doxycycline-inducible lentiviral vector [41] (Fig. 6A). In the absence of doxycycline, Trk protein expression was below the detection limit (Fig. 6B, S7). However, induction of expression with 1 µg/ml doxycycline for 48 h led to the constitutive activation of TrkB or *SQSTM1-NTRK2* (Figs. 6B, S7). Another interesting observation was that the *SQSTM1-NTRK2* protein, with a predicted molecular weight of 61 kDa, appeared at approximately 60 kDa and 120 kDa under standard SDS-PAGE Western blotting conditions, indicating that the protein tends to form stable SDS-resistant dimers (Fig. 6B). TrkB was strongly enriched close to F-actin-rich protrusions (Fig. S7), arrows in magenta). Notably, immunoreactivity was observed in two fractions of pTrk-kin. The most prominent pTrk-kin signal was close to the perinuclear region in Golgi apparatus-like localization. The signal was negative for the receptor domain antibody (Fig. S7B, C, and cyan arrows). The distribution pattern of TrkB and F-actin was reminiscent of the typical cell morphology of non-migrating cells [68].

Subsequently, U87MG cells expressing different Trk constructs were seeded into 2-well silicon inserts with a defined cell-free gap to assess random migration (Fig. 6C). For cell seeding, we added 1 µg/ml doxycycline to induce the expression of the three constructs. After another 24 h, we removed the silicon insert and observed whether the induction of TrkB accelerated or slowed down random cell migration. The experiment showed that expression induction significantly reduced the migration of TrkB-wt expressing cells compared to that of TrkB-YFF-expressing cells (Fig. 6C, D). Notably, *SQSTM1-NTRK2* expressing cells exhibited pronounced morphological alterations and highly impaired migration (Figs. 6C, D, and S7). The cells were roundish, their migration was weak, and cell clone formation was observed. The SQSTM1-fusion domain carries the PB1 domain which might promote dimerization [12, 65]. Despite these effects, the induced U87MG cells expressing TrkB-wt, TrkB-YFF, or SQSTM1-NTRK2 did not die off immediately (Fig. S7). We also performed Western blotting against phospho-caspase 3 (Ser^150^) but did not observe activation of this apoptosis marker.

### Kinase-dependent, transcriptional reprogramming of the cell phenotype

The rapid onset of phenotypic changes upon the expression of TrkB kinase or *SQSTM1-NTRK2* prompted the question of whether self-active kinase signaling also induces global alterations in the gene expression profile. To address this, we performed a comparative analysis of the transcriptomes of U87MG cells under different experimental conditions (four biological replicates per condition; total number of transcriptomes: 36; GEO accession: GSE186207, Table 4). We controlled for lentiviral infection, doxycycline treatment, abundance, and kinase activity.

**Table 4:** Transcriptomes of U87MG cells under different experimental conditions.

Condition	GEO sample (GSE186207)	Comment
U87MG	line_ctr (1–4)	Cell line control
U87MG TrkB-wt	wt_ctr (1–4)	*TrkB expression is silenced*.
U87MG TrkB-wt (Dox)	wt_dox (1–4)	*TrkB expression, self-active signaling is induced*.
U87MG TrkB-YFF	YFF_ctr (1–4)	*TrkB-YFF expression is silenced*.
U87MG TrkB-YFF (Dox)	YFF_dox (1–4)	*TrkB expression is induced, but self-active signaling is interrupted by mutating Y*^*705*^*/Y*^*706*^.
U87MG-*SQSTM1-NTRK2*	fus_ctr (1–4)	*NTRK2-fusion expression is silenced*.
U87MG-*SQSTM1-NTRK2* (Dox)	fus_dox (1–4)	*NTRK2-fusion expression, self-active signaling is induced*.
U87MG TrkB-wt (Dox), LOXO-101 treated	wt_loxo (1–4)	*TrkB expression, but kinase signaling is acutely blocked by a kinase inhibitor*.
U87MG-*SQSTM1-NTRK2* (Dox), LOXO-101 treated	fus_loxo (1–4)	*NTRK2-fusion expression, but kinase signaling is acutely blocked by a kinase inhibitor*.

**Fig. 7:**
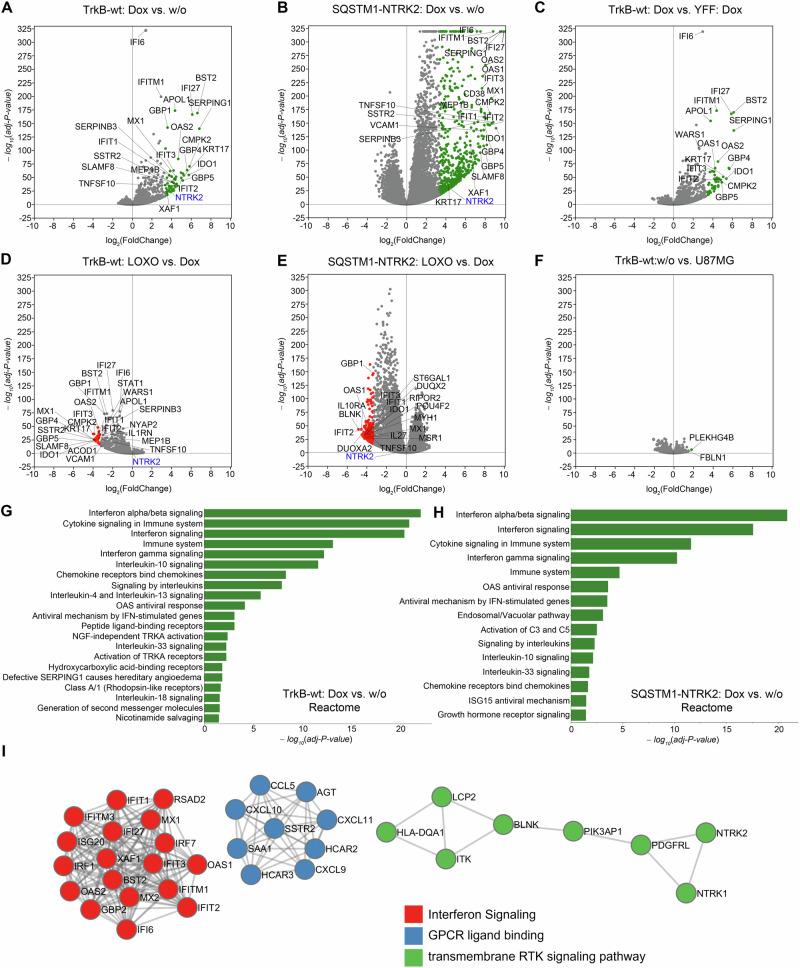
Constitutive kinase signaling of TrkB and SQSTM1-NTRK2 activates a specific transcriptome. **A**–**F** RNA-seq analysis of U87MG cells stably expressing either TrkB-wt, TrkB-YFF, or the fusion protein SQSTM1-NTRK2 via the Tet-on system: Volcano plots showing genes that were upregulated (right half) or downregulated (left half) depending on 48 h induction of TrkB (Dox) or upon further 24 h treatment with LOXO-101 (Loxo). Log2-fold-change values were plotted as *P*-adjusted values obtained from DESeq2 analysis of the transcriptome. Furthermore, the top 10–20 of these, up or down-regulated genes (and NTRK2) have been labeled (**A**–**F**). As a rule, up-regulated genes can be found on the right half of the volcano plots and down-regulated on the left. In (**F**), TrkB-wt (uninduced control) was compared to the U87MG cell line to eliminate possible underlying effects arising from the cell line or from the lentiviral infection itself. **G**, **H** Reactome analysis revealed a strong enrichment of genes involved in cellular immune defense responses, such as those required for interferon, chemokine, and interleukin signaling. Reactome with the top 20 biological pathways. Intracellular TkB kinase self-activation-induced immune responses (in **G**) overlap with those of the SQSTM1-NTRK2 fusion (in **H**). **I** Protein-protein interaction (metascape analysis) of upregulated genes found in TrkB-wt (Dox) as well as SQSTM1-NTRK2 (Dox). Data filtering: log2-fold upregulation, *p*-adjusted value < 0.05.

TrkB caused transcriptional reprogramming of U87MG cells by upregulating various immune response genes such as BST2, SERPNG1, IFI27, IDO1, GBP4, KRT17, IFIT2, IFI6, and TNSF10 (Fig. 7). We found 55 genes showing a 10-fold and 517 genes showing a 2-fold increase in expression levels. Reactome analysis [53] using g:Profiler [52] revealed a strong enrichment of genes involved in immune defense responses, such as those required for interferon, chemokine, and interleukin signaling (Fig. 7). These similar genes and pathways were downregulated upon acute blockade of TrkB kinase activity with LOXO-101 Fig. 7).

The impact of *SQSTM1-NTRK2* fusion on gene expression was even more pronounced. We observed a significantly higher number of upregulated genes, with 626 genes exhibiting a 10-fold increase and 3883 genes showing a 2-fold increase in expression (Fig. 7). Importantly, when we expressed the TrkB-YFF mutant, which lacks kinase activity, we did not observe the same transcriptome changes induced by wild-type TrkB or *SQSTM1-NTRK2* fusion (Figs. 7, S8). To ensure the reliability of our experimental conditions, we included additional vector control conditions that confirmed the tight regulation of TrkB expression and lentivirus safety (Figs. 7, S8).

Transcripts upregulated by TrkB-wt (log2-fold, padj < 0.05) were also found to be upregulated in the SQSTM1-NTRK2 transcript. A protein-protein interaction analysis identified three protein interaction clusters. One for interferon signaling, one for G-protein coupled receptor ligand binding and one for receptor tyrosine kinase signaling (Fig. 7). Of note, expression of the proliferation marker Ki67 (gene: MKI67) was 3.3-fold downregulated by SQSTM1-NTRK2 expression (ctr: 125 TPM; DOX-induced: 37 TPM, mean values of *n* = 4) (see supplementary TPM count table). Transcriptome changes by SQSTM1-NTRK2 could not fully be reverted with LOXO-101, indicating a contribution of the SQSTM1/p62 domains to the cell phenotype.

### Cell transformation in NIH3T3 cells

For the Trk fusion protein *ETV6-NTRK3* it was shown that it can transform NIH3T3 cells [39], a cell model to investigate oncogenic activity of overexpressed genes. We were asked whether atypical activation of TrkB would also transform NIH3T3 cells. To answer this question, we infected NIH3T3 cells with our doxycycline-inducible lentiviral vectors and selected stable cell lines with high concentrations of puromycin (2 µg/ml). In the absence of doxycycline, TrkB or SQSTM1-NTRK2 protein expression was below the detection limit (Fig. S9). However, induction of expression with 1 µg/ml DOX for 72 h led to the constitutive activation of *SQSTM1-NTRK2*, but not TrkB-wt. In the NIH3T3 cells, in contrast to HEK293 or U87MG, TrkB-wt appeared exclusively at 130 kDa, indicating full maturation. To confirm that TrkB-wt in NIH3T3 cells is functional, we stimulated the stable cell line with 20 ng/ml BDNF. Cells were not serum depleted. BDNF-induced phosphorylation of TrkB within 15 min (Fig. S9). We also monitored the cell morphology of NIH3T3 cells with classical assays, typically used to test for NIH3T3 cell transformation (Fig. S9). Neither in soft agar assays nor in plate assays we could see foci formation, neither by TrkB-wt nor by *SQSTM1-NTRK2*. We also tested constitutive activation of Myr-ICD and ICD-expressing cell lines, but both did not become constitutive active under DOX-on conditions. In contrast to TrkB, SQSTM1-NTRK2 caused changes in cell morphology (size and vacuolization) (Fig. S9). To validate the data, we performed immunolabeling experiments. Also, these data confirmed the lack of constitutive activation of TrkB-wt, but cell morphology changes by constitutive active SQSTM1-NTRK2 (Fig. S9).

### TrkB kinase in human glioblastoma samples

As all Trk receptors show expression-dependent activation, we asked for evidence of atypical phospho-Trk in brain tumor samples. We recently showed a trend for reduced survival in patients with high levels of Nestin, a neural stem cell marker, in glioblastoma tumor samples [69]. High Nestin expression can also be used to distinguish glioma cells from unaffected brain tissue with a high probability [70, 71]. Furthermore, TrkB kinase is active in Nestin+ adult neural stem cells [72]. Due to these indications, we asked for evidence of atypical Trk kinase signals in Nestin+ glioblastoma tissue from our local tissue bank [69] (sample description: Table S2).

**Fig. 8:**
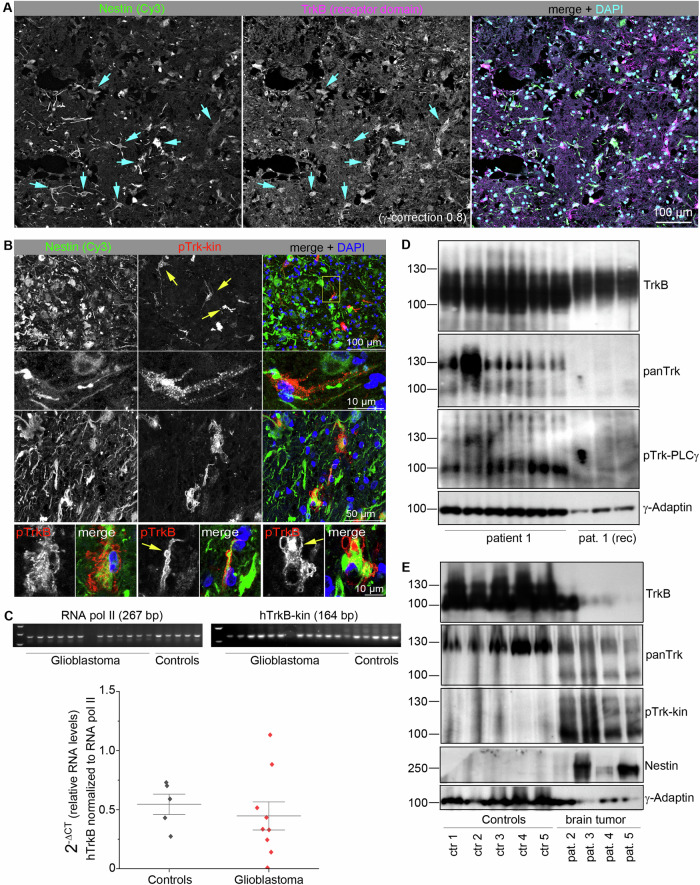
Localization and expression of TrkB, panTrk and pTrk in grade IV glioblastoma. **A** Nestin/TrkB-positive cells in glioblastoma. Sections were labeled for anti-Nestin, to identify glioblastoma, and the TrkB receptor. Confocal laser scanning images. DAPI was used as the counterstain. Some Nestin+ cells show a high abundance of TrkB (cyan arrows). **B** Immunolabeling of representative glioblastoma cryosections. with anti-Nestin and anti-pTrk-kin. Single Nestin+ cells show a high abundance of pTrk-kin (upper panel, yellow arrows). Second and third panels: High-resolution confocal image stack showing pTrk in intracellular, immunoreactive clusters. Lower panel. Membrane bleb-like structures (yellow arrow) in single cells with strong intracellular pTrk-kin label. **C** RT-qPCR revealed abundant expression of TrkB kinase transcript in grade IV glioblastoma. Normalization of TrkB kinase expression levels in relation to RNA polymerase II. Single data points, mean values, and standard deviations are shown. The amplicon sizes were verified by agarose gel electrophoresis, as indicated. **D** Immature phospho-active Trk kinase in glioblastoma. Western blotting of whole-cell lysates generated from frozen post-mortem glioblastoma samples with the indicated antibodies. Lanes 1–6 show the lysates generated from different tissue pieces of the same glioblastoma sample. Note the high abundance of panTrk kinase and pTrk in one tissue sample (lane 2). Lane 7–9 shows representative TrkB-positive, panTrk kinase-negative, pTrk-kin-negative glioblastoma samples. This sample (pat. 1 (rec)) is a recurrent cancer sample from the same patient. γ-Adaptin was labeled as loading control. **E** TrkB and phospho-active Trk kinase in glioblastoma cells. Western blotting of whole-cell lysates from human brain or glioblastoma samples. Lanes 1–5 show control brain samples (frontal brain). PanTrk kinase immunoreactivity was observed at 130 kDa, indicating mature Trk (either TrkA, B or C). Lanes 6–9 show total lysates from different grade IV glioblastoma biopsies. Note the high abundance of panTrk kinase and pTrk-kin at approximately 90 kDa, indicating immature, phosphorylated Trk. For all the lanes, 40 µg of total protein was blotted.

To localize pTrk in Nestin+ glioblastoma tissue, we performed immunofluorescence labeling with anti-Nestin. Morphologies ranged from Nestin+ clone-like cell clusters, cells rich in neurites, cell clumps, or densely packed cell masses (Fig. S10). Cryosections were then labeled with anti-Nestin [69] and anti-TrkB. In single samples, TrkB appeared in Nestin+ cells, at least in certain areas of glioma tissue (Fig. 8A). High-resolution z-stack confocal microscopy of occasional pTrk-positive cells showed single Nestin+ cells carrying intracellular pTrk-positive clusters and pTrk-positive bleb-like formations (Fig. 8B).

To distinguish the expression of TrkB kinase from that of TrkB-T1, we performed reverse transcriptase qPCR (Fig. 8C). The upper primer was designed to target an exon encoding the receptor domain, whereas the lower primer was bound to an exon encoding the kinase domain (details provided in Table 3). A rather high expression level of the TrkB kinase transcript was detected in the control samples of the frontal brain tissue (approximately 50% of the housekeeping gene RNA polymerase II, Fig. 8C). In glioblastoma, a rather high but variable expression of TrkB kinase was observed (Fig. 8C).

Due to the strong immunoblotting signal obtained with anti-TrkB antibodies, it was difficult to clearly resolve the 90 and 130 kDa bands corresponding to TrkB isoforms (Fig. 8D). To address this, we reprobed the blots with anti-panTrk, an antibody that detects TrkA, TrkB, and TrkC (Table S1). This verified the expression of Trk kinase at 90 kDa (immature Trk) and 130 kDa (mature Trk) (Fig. 8D). Subsequent probing with anti-pTrk-PLCγ confirmed pTrk at 90 kDa, suggesting phosphorylation of an immature Trk kinase, either TrkA, TrkB, or TrkC (Fig. 8D). In one of the lysates, the 130 kDa band also showed an anti-pTrk signal (Fig. 8D). In the recurrent tissue samples (pat. 1 (rec)), neither panTrk kinase nor pTrk could be detected (Fig. 8D). Thus, the recurrent tumor sample expressed the truncated TrkB-T1, detected with anti-TrkB, but no detectable levels of any Trk kinase isoform.

**Fig. 9:**
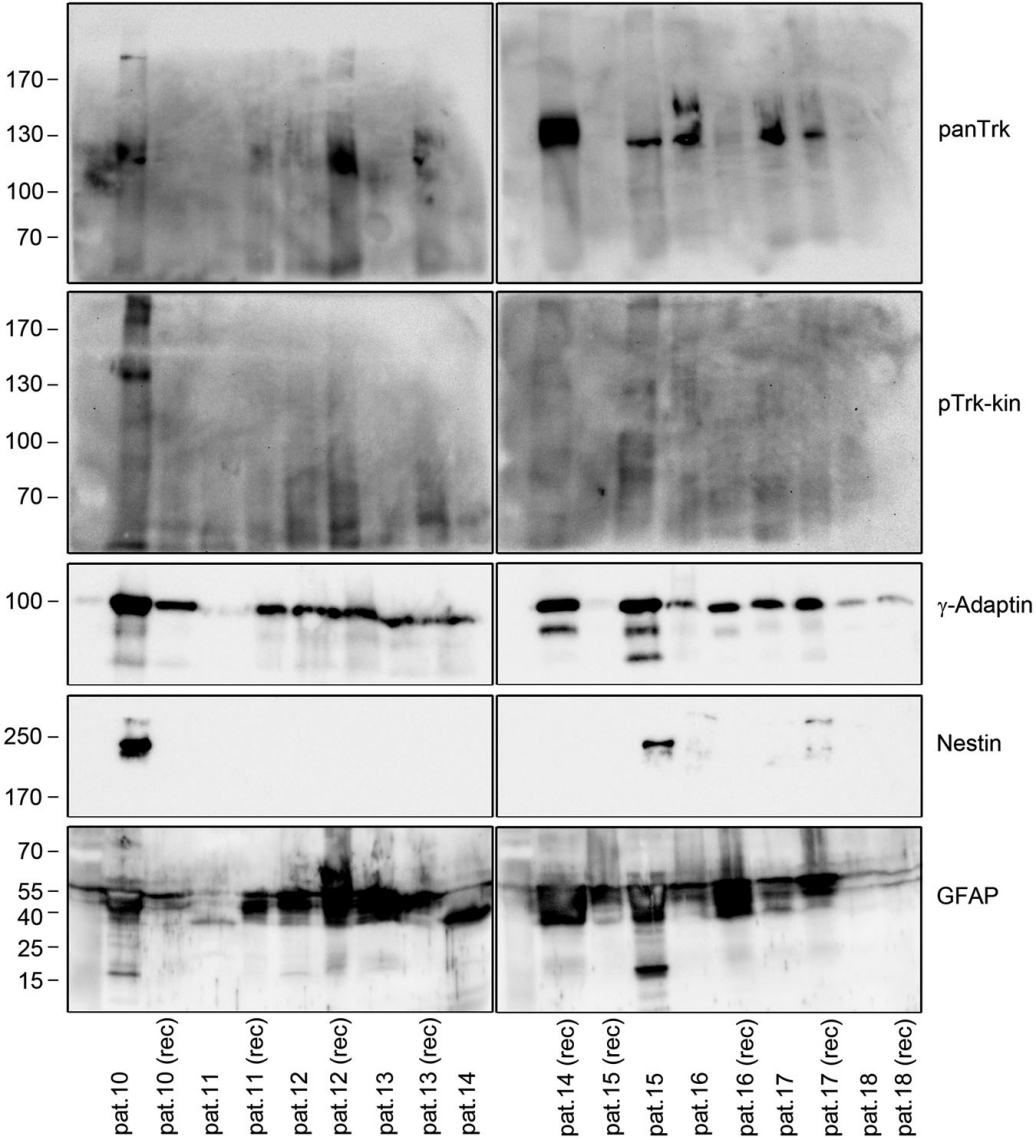
Expression of pTrk-kin in grade IV glioblastoma. Trk and phospho-active Trk kinase in glioblastoma. Western blotting of whole-cell lysates (40 µg per sample) generated from frozen glioblastoma samples with indicated antibodies. Total protein lysates from indicated tumor tissue pieces (see Table S2) were investigated. Note anti-pTrk-kin signals at 130 kDa in Nestin+ samples (in lanes for pat.10 and pat.15). In one recurrent sample (pat.14 (rec)), a strong anti-panTrk immunoreactivity appeared.

Next, we compared protein lysates from the human frontal brain with those from different Nestin+ brain tumor samples (first diagnosis samples) (Fig. 8E). In these samples, we observed pronounced TrkB, e.g. in patient 2, and pTrk kinase signals for all samples. Notably, the 90 kDa panTrk kinase signal was exclusively observed in brain tumor samples. In frontal brain controls, neither the 90 kDa Trk-kinase, nor any pTrk signals were detected (Fig. 8E). The data suggest that at least one of the three Trk receptors is immature and phospho-active in this specific Nestin+ tumor sample. Furthermore, we expanded our investigation to include additional glioblastoma samples (Fig. 9, Table S2). These tumor biopsies had not undergone pre-examination by a neuropathologist to confirm Nestin expression [69]. Two samples were Nestin/pTrk-kin-positive. We did not find pTrk-kin in recurrent tumors (*n* = 10).

The data show that a combination of panTrk and anti-pTrk antibodies is useful for screening tumor samples for atypical Trk activation. As a diagnostic tool, this approach has the potential to detect atypical *NTRK* signaling and the activity of *NTRK*-fusion proteins harboring an active Trk kinase domain in suspected tumor biopsies.

## Discussion

This study shows atypical biological effects caused by constitutive activation of the TrkB proto-oncogene. These data provide a biological explanation for the ligand-independent signaling of intracellular Trk kinase domains and intracellular NTRK-fusion oncogenes. Importantly, effects downstream of active kinase domains are effectively blocked by clinically approved Trk kinase inhibitors, like Larotrectinhib or Entrectinhib. As the kinase domain of Trk family members is highly conserved, it is possible that the observed signaling principles hold true for all Trk family members and other kinase fusion proteins.

### Trk autophosphorylation versus neurotrophin-independent transactivation

The isolated ICD of TrkB can undergo abundance-dependent autophosphorylation [18, 19]. Albeit we cannot exclude that any other RTK transactivates intracellular TrkB kinase domains by a yet unknown mechanism, we assume that overexpression and high abundance of ICD domains enable transactivation and autophosphorylation between ICDs. This interaction in trans might be transient because TrkB ICDs seem to have a less pronounced tendency to form stable dimers than TrkA ICDs [18]. Critical for the atypical signaling is Y^705^ in TrkB. In silico phosphorylation of Y^705^ or the Y^705^F mutation results in a structural shift in the corresponding kinase domain. This transition could be indicative of the concept that release from cis-autoinhibition initiates Y^705^-mediated signaling. This suggests that a pharmacologic, small-molecule block of the release from cis-autoinhibition at Y^705^ or the stabilization of the YFY-like confirmation would be an interesting strategic option to block constitutively active *NTRK* in cancer, for instance, in the case of acquired resistance to prior Trk kinase inhibition [73, 74]. In this context, it is important to note that phospho-site substitution of Y^705^ with glutamate (E) or aspartate (D) does not mimic atypical signaling to pFAK. This is another hint that Y705 phosphorylation is critical for the initiation and maintenance of intracellular, constitutive signaling effects.

Functional interaction between EGF receptor (EGFR) and TrkB has been demonstrated [16, 23, 75, 76]. Two studies show that EGF-induced transactivation of TrkB promotes cell migration [16, 76]. This effect is different from our observations as atypical TrkB signaling cannot be blocked by serum depletion and does not promote cell migration. Furthermore, EGF-mediated transactivation (trans-signaling) can be blocked with the Src-inhibitors PP1 and PP2 [16], which is not the case for atypical signaling. It was also shown that ANA-12, a TrkB receptor domain blocker, can reduce the viability of U87MG cells [75]. When ANA-12 was applied together with the EGFR inhibitor AG1478, a synergistic effect of combined TrkB/EGFR inhibition was observed on cell viability in vitro, but not in an intracranial U87MG xenograft model. In our experiments, atypical activation of TrkB was BDNF-independent and ANA-12-independent, indicating that atypical activation is different from the effects described by Pinheiro et al. [75].

### Autoactivation of TrkB ICDs – an alternative for constitutive signaling by Y^705^

Downstream signaling of TrkB typically occurs via TIAM/Rac -, Shc-adapter -, or PLCγ/Ca^2+^-dependent signaling cascades [6, 7, 12]. The critical role of Y^705^ ( = Y^706^ in humans) in the tyrosine triplet is not surprising, as previous studies have highlighted the importance of this evolutionarily conserved tyrosine in the Trk family [8, 10, 12, 19, 20].

More surprising are the following observations:

1. The kinase domain becomes active at intracellular sites, without receptor, transmembrane, or juxtamembrane domains, in the absence of neurotrophins or serum components.
2. The persistent activity of intracellular Y^705^ leads to specific, reversible cellular effects.
3. Atypical signaling of TrkB and the SQSTM1-NTRK2 oncogene are anti-migratory, while BDNF/TrkB signaling in cancer seems to be promigratory [12, 66].
4. Trk kinase inhibitors, but not by receptor inhibitors, acutely block atypical functions of Trk.

Thus, ICD-mediated constitutive activation represents a distinct category of TrkB kinase domain signaling (*self-active Trk kinase domain signaling*).

### Constitutive active TrkB kinase in grade IV glioblastoma?

In human grade IV glioblastoma tissue, we found marked differences in TrkB characteristics when compared to frontal brain control tissue. These results do not exclude the possibility that the natural TrkB ligand BDNF, from neurons, microglia, serum [77] or platelets [78] stimulates Trk phosphorylation. However, previous studies have shown that the recombinant expression of a TrkB construct lacking the BDNF-binding domain is sufficient to confer an aggressive carcinogenic phenotype to a neural crest-derived cell line [79]. Thus, it is plausible that natural TrkB could contribute disadvantageous signals in the absence of neurotrophins. Given the availability of approved *NTRK* inhibitors, clinical studies could explore the potential of anti-Trk treatment to improve outcomes in glioblastoma patients with pronounced atypical pTrk signals. Western blot-like immunoassays are well suited for identifying molecular size shifts of *NTRK*-fusion proteins (and corresponding dimers) (see Fig. 6C) and underglycosylated (intracellular) active Trk receptor kinases (see Fig. 8, compare with Fig. 2E in Lawn et al. [23]). For this purpose, pan-Trk antibodies and anti-phospho-Trk-kin antibodies would be most helpful.

Neutrotrophin signaling via endogenous TrkB and TrkC has been shown to promote the in vitro growth and viability of brain tumor-initiating cells, isolated from human glioblastoma biopsies [23]. Notably, this effect was fully neurotrophin-dependent and also observed in the absence of serum and growth factors such as EGF and FGF. In the context of our data, it indicates that both signaling pathways, atypical and classical signaling might coexist in glioblastoma. It can well be that the cell surface fraction of Trk receptors in glioblastoma is neurotrophin-dependent, while immature or intracellular Trk activates atypical signaling.

## Limitations

1. Signaling investigation relies on the heterologous expression of TrkB kinase, TrkB mutants, theoretical kinase constructs (e.g., Myr-ICD/ICD), and a representative NTRK2-fusion (deduced from RNA-seq data). Currently, cell lines carrying NTRK2-fusions are not available, and commonly used glioblastoma-like cell lines such as U87MG, which were previously suggested to express functional TrkB protein [75], did not show much Trk expression (see Supplementary Information – TPM count table with RNAseq data: control U87MG cells express 0.25 transcripts per million of TrkB) or panTrk. Therefore, we used heterologous expression in cell lines (HEK293, NIH3T3, U87MG) to describe the cellular responses of atypically active Trk kinase.
2. It should be noted that the activation of focal adhesion kinase (FAK) by overexpressed TrkB was observed only in HEK293 cells. We could not detect pFAK in the glioblastoma tissue. Anyhow, we think the data is novel and worth reporting. It can well be that pFAK activation can become a biomarker for the signaling activity of solely cytosolic NTRK-fusion proteins in tumors in the future.
3. We did not test these critical constructs in vivo. However, TrkB kinase overexpression in the rodent brain has already been demonstrated by others [80] and there is no justifiable argument for repeating this burdensome animal experiment (3 R principle).
4. We performed a retrospective investigation of the existing glioblastoma samples. We hope that the data reported herein will pave the way for more systematic examinations of tumor samples on atypical Trk using western blotting (ethical approval and financial support).
5. We could not provide systematic single-cell transcriptome data. In the context of this study, single-cell profiling would make sense in Nestin-positive glioblastoma tissue with a high abundance of pTrk-kin at 90 kDa (immature Trk), or in the case of kinase-active NTRK-fusion proteins in a corresponding cancer biopsy. Such a study might help determine the extent to which constitutive active Trk kinases correlate with cell-specific reprogramming phenotypes or specific cell surface antigens.

## Supplementary information

Video 1. Time-lapse video showing GFP-actin dynamics of HEK293 cells expressing TrkB-wt

Video 2. Time-lapse video showing GFP-actin dynamics of HEK293 cells expressing TrkB-wt

Video 3. Time-lapse video showing GFP-actin dynamics of HEK293 cells expressing TrkB-YFF

Video 4. Time-lapse video showing GFP-actin dynamics of HEK293 cells expressing TrkB-YFF

Supplemental material - Gupta et al (2024)

## Data Availability

The data generated in this study are available upon request from the corresponding authors. RNA-seq data can be found in the GEO accession: GSE186207. The corresponding TPM tables are provided in the supplementary information. Unprocessed Western blots are shown in supplementary information. The clones and raw image data are available upon request.
