# Supplementary material for: Atypical cellular responses mediated by intracellular constitutive active TrkB (NTRK2) kinase domains and a solely intracellular NTRK2-fusion oncogene
Source: Cancer Gene Ther. 2024 Jul 22;31(9):1357–79. doi: 10.1038/s41417-024-00809-0 (PMC11405271; doi:10.1038/s41417-024-00809-0)
Supplement: Supplementary file 5 — Supplemental material - Gupta et al (2024) [file 41417_2024_809_MOESM5_ESM.pdf]

SUPPLEMENTARY MATERIAL

Atypical cellular responses mediated by intracellular constitutive active TrkB (NTRK2) kinase domains and a solely intracellular NTRK2-fusion oncogene

Rohini Gupta, Melanie Dittmeier, Gisela Wohlleben, Vera Nickl, Thorsten Bischler, Vanessa Luzak, Vanessa Wegat, Dennis Doll, Annemarie Schulte, Elena Bady, Georg Langlhofer, Britta Wachter, Steven Havlicek, Jahnve Gupta, Evi Horn, Patrick Lüningschrör, Carmen Villmann, Bülent Polat, Jörg Wischhusen, Camelia M. Monoranu, Jochen Kuper, & Robert Blum

Corresponding author: Robert Blum, University Hospital Würzburg, Department of Neurology, Würzburg, Germany. Email: Blum_R@UKW.de

Suppl. data

suppl. mat #2: transcriptome data (TPM normalization of RNA-seq raw data)

Figures, Videos, and tables

table S1. Properties of anti-Trk antibodies

antibody	TrkB wt (western)	TrkB wt (ICC)	TrkB-ATP mut. (western)	TrkB-ATP mut. (ICC)	human tissue	Comment: properties (in our hands)
panTrk (C-term) – A7H6R (anti-panTrk)	+	+	+	+	+	• detected recombinant TrkA, TrkB, TrkC
anti-TrkB (receptor domain) (anti-TrkB)	+	+	+	+	+	• detected TrkB kinase at 130kDa (glycosylated) and 90 kDa<br>• detected recombinant TrkB-T1
anti-pY674/675-TrkA (anti-pY706/707–TrkB) C50F3 (anti-pTrk-kin)	+	+	-	-	+	• detected YYF mutant, did not detect YFY, YDY, YEY mutants<br>• did not detect recombinant pTrkC
pY490-TrkA (anti-pY516-TrkB) C35G9 (anti-pTrk-shc)	+	+	-	-	+	• detected Shc mutant $Y^{515}F$ in ICC, but not in Western
pY785-TrkA (anti-pY816-TrkB) C67C8 (anti-pTrk-PLCγ)	+	+	-	-	+	• detected PLCγ mutant $Y^{816}F$ in ICC<br>• did not detect pTrkC<br>• immunoreactive when HEK293 cells where treated with 10µg/ml EGF for 15 min (presumably cross-reactive to human pEGF receptor)

Table S2: Information: Brain tumor tissue samples. Tissue represents glioblastoma, IDH wildtype, CNS WHO grade 4. The patient 2 biopsy was categorized as astrocytoma, IDH-mutant, CNS, WHO grade 4.

sample	tissue code	investigation	pTrkB-kin	age / sex	comment
**tissue samples (Fig. 1): Nestin+ in histological pre-examination (Polat et al., 2022)**					
1.	Patient 1	WB / qPCR	+	55 / male	temporal le, first diagnosis
2.	Patient 1-rec	WB		recurrent glioblastoma of patient 1	
3.	Patient 2	WB / qPCR	+	44 / female	frontal ri, relapse
4.	Patient 3	WB / qPCR	+	61 / male	frontal le, first diagnosis
5.	Patient 4	WB / qPCR	+	60 / male	postcentral le, first diagnosis
6.	Patient 5	WB / qPCR	+	67 / male	central ri, first diagnosis
7.	Patient 6	qPCR	n.d.	71 / male	frontal le, first diagnosis
8.	Patient 7	qPCR	n.d.	80 / male	temporal ri, first diagnosis
9.	Patient 8	qPCR	n.d.	52 / female	temporal le, first diagnosis
10.	Patient 9	qPCR	n.d.	51 / male	occipital ri, first diagnosis
**tissue samples (Figure S12): available samples randomly selected**					
11.	Patient 10	WB	+	43 / female	43, f, temporal ri, first diagnosis
12.	Patient 10-rec	WB		recurrent glioblastoma of patient 10	
13.	Patient 11	WB		65 / female	65, f, temporal le, first diagnosis
14.	Patient 11-rec	WB		recurrent glioblastoma of patient 11	
15.	Patient 12	WB		67 / male	temporal ri, first diagnosis
16.	Patient 12-rec	WB		recurrent glioblastoma of patient 12	
17.	Patient 13	WB		68 / female	temporal ri, first diagnosis
18.	Patient 13-rec	WB		recurrent glioblastoma of patient 13	
19.	Patient 14	WB		69 / male	frontal le, first diagnosis
20.	Patient 14-rec	WB		recurrent glioblastoma patient 14	
21.	Patient 15-rec	WB		recurrent glioblastoma of patient 15	
22.	Patient 15	WB	+	44 / female	temporal le, first diagnosis
23.	Patient 16	WB		60 / male	temporal le, first diagnosis
24.	Patient 16-rec	WB		recurrent glioblastoma of patient 16	
25.	Patient 17	WB		45 / female	temporal ri, first diagnosis
26.	Patient 17-rec	WB		recurrent glioblastoma patient 17	
27.	Patient 18	WB		64 / female	craniocervical junction, first diagnosis
28.	Patient 18-rec	WB		recurrent glioblastoma of patient 18	
**Frontal cortex tissue samples**					
29.	Control 1	WB / qPCR		51 / female	advanced anal carcinoma, end stage
30.	Control 2	WB / qPCR		72 / female	heart infarction/attack
31.	Control 3	WB / qPCR		33 / female	EBV-assoc. hemophagocytic syndrome
32.	Control 4	WB / qPCR		70 / male	mesenteric infarction
33.	Control 5	WB / qPCR		70 / male	pancreatic carcinoma

** Polat, B., G. Wohlleben, R. Kosmala, D. Lisowski, F. Mantel, V. Lewitzki, M. Lohr, R. Blum, P. Herud, M. Flentje, and C.M. Monoranu. 2022. Differences in stem cell marker and osteopontin expression in primary and recurrent glioblastoma. Cancer Cell Int. 22:87.* Abbreviations: le – left; ri – right; WB – Western blot.

Videos

Video 1. Time-lapse video showing GFP-actin dynamics of HEK293 cells expressing TrkB-wt.

Overview. Time-lapse created from confocal x.y-t image series. Scale bar 25 µm.

Video 2. Time-lapse video showing GFP-actin dynamics of HEK293 cells expressing TrkB-wt.

Detail of membrane blebbing. Time-lapse created from confocal x.y-t image series. Scale bar 25 µm.

Video 3. Time-lapse video showing GFP-actin dynamics of HEK293 cells expressing TrkB-YFF.

Overview. Time-lapse created from confocal x.y-t image series. Scale bar 25 µm.

Video 4. Time-lapse video showing GFP-actin dynamics of HEK293 cells expressing TrkB-YFF.

Detail of filopodia dynamics. Time-lapse created from confocal x.y-t image series. Scale bar 25 µm.

A) Mouse TrkB (reference sequence NM_001025074; NP001020245)

```
  1 MSPWLKWHGP AMARLWGLCL LVLGFWRASL ACPTSCKCSS ARIWCTEPSP GIVAFPRLEP
                signal peptide                +1
 61 NSVDPENITE ILIANQKRLE IINEDDVEAY VGLRNLTIVD SGLKFVAYKA FLKNSNLRHI

121 NFTRNKLTSL SRRHFRHLDL SDLILTGNPF TCSCDIMWLK TLQETKSSPD TQDLYCLNES

181 SKNMPLANLQ IPNCGLPSAR LAAPNLTVEE GKSVTLSCSV GGDPLPTLYW DVGNLVSKHM

241 NETSHTQGSL RITNISSDDS GKQISCVAEN LVGEDQDSVN LTVHFAPTIT FLESPTSDHH

301 WCIPFTVRGN PKPALQWFYN GAILNESKYI CTKIHVTNHT EYHGCLQLDN PTHMNNGDYT

361 LMAKNEYGKD ERQISAHFMG RPGVDYETNP NYPEVLYEDW TTPTDIGDTT NKSNEIPSTD

421 VADQSNREHL SVYAVVVIAS VVGFCLLVML LLKLARHSK FGMKGPASVI SNDDDSASPL
               transmembrane domain         start ICD (intracellular kinase domain)   +478
481 HHISNGSNTP SSSEGGPDAV IIGMTKIPVI ENPQYFGITN SQLKPDTFVQ HIKRHNIVLK
                                        +515 (Shc)
541 RELGEGAFGK VFLAECYNLC PEQDKILVAV KTLKDASDNA RKDFHREAEL LTNLQEHEIV
                                    +571 (ATP-binding)
601 KFYGVCVEGD PLIMVFEYMK HGDLNKFLRA HGPDAVLMAE GNPPTELTQS QMLHIAQQIA

661 AGMVYLASQH FVHRDLATRN CLVGENLLVK IGDFGMSRDV YSTDYYRVGG HTMLPIRWMP
                                                YxxxYY (tyrosine triplett)
721 PESIMYRKFT TESDVWSLGV VLWEIFTYGK QPWYQLSNNE VIECITQGRV LQRPRTCPQE

781 VYELMLGCWQ REPHTRKNIK SIHTLLQNLA KASPVYLDIL G
                                        +816 (PLCγ)
```

Fig. S1. TrkB model and construct. A. Deduced amino acid sequence of *Ntrk2* (*trk*B full-length – *trk*B.FL) encoded by *Mus musculus* (reference: NM_001025074; NP_001020245). In the depicted amino acid sequence, in blue is the initiating methionine and signal peptide, in orange is the transmembrane domain, in purple the lysine residue of the ATP binding site and in green are the important serine or tyrosine residues of the kinase domain. Putative N-glycosylation sequons are indicated in red and yellow.

constructs	synonym	Features, signaling pathway regulation
TrkB - wt	wildtype	Wildtypic TrkB, BDNF / NT4/5 receptor
TrkB – S^{478}A	TrkB – S^{478}A	Interaction to TIAM1 / Rac1 pathway
TrkB - Y^{515}F	Shc mutant	Shc adaptor site, Ras / MAPK pathway, Pi3K / Akt pathway
TrkB – K^{571}N	ATP mutant	ATP-binding, kinase-dead mutant
TrkB – Y^{705}F	YFY mutant	YxxYY motif mutant, critical mutation in the in the activation loop
TrkB – Y^{706}F	YYF mutant	YxxYY motif mutant mutant, kinase-active
TrkB – Y^{705,706}F	YFF mutant	YxxYY motif double mutant
TrkB – Y^{705}D	YDY mutant	Phospho-mimicking mutation YxxYY motif
TrkB – Y^{705}E	YEY mutant	Phospho-mimicking mutation YxxYY motif
TrkB – Y^{816}F	PLCγ mutant	Adaptor site for PLCγ / IP_3 / calcium pathway
TrkB - Y^{515}F, Y^{816}F	Shc-PLCγ double mutant	Shc adaptor site and PLCγ–site are mutated
TrkB – K^{571}N, Y^{705}D	YD-ATP double mutant	Double mutant: phospho-mimicking mutation in YxxYY motif, and kinase-dead mutation at K^{571}
TrkB – K^{571}N, Y^{705}E	YD-ATP double mutant	Double mutant: phospho-mimicking mutation in YxxYY motif, and kinase-dead mutation at K^{571}
TrkB-Myr ICD	Myr-ICD	Intracellular domain: K^{454} – STOP, membrane anchored by N-terminal myristoylation motif
TrkB-ICD	ICD	cytosolic, intracellular domain K^{454} – STOP
TrkB-4×N^{mut}A	TrkB^{4gly}	Mutation of 4 predicted N-glycosylation sites
TrkB-7×N^{mut}A	TrkB^{7gly}	Mutation of 7 predicted N-glycosylation sites
TrkB-12×N^{mut}A	TrkB^{12gly}	Mutation of 12 predicted N-glycosylation sites
SQSTM1-*NTRK2*	SQSTM1-NTRK2	SQSTM1-NTRK2 kinase fusion construct

Fig. S1. B. Table explaining the various TrkB constructs generated for use in this study.

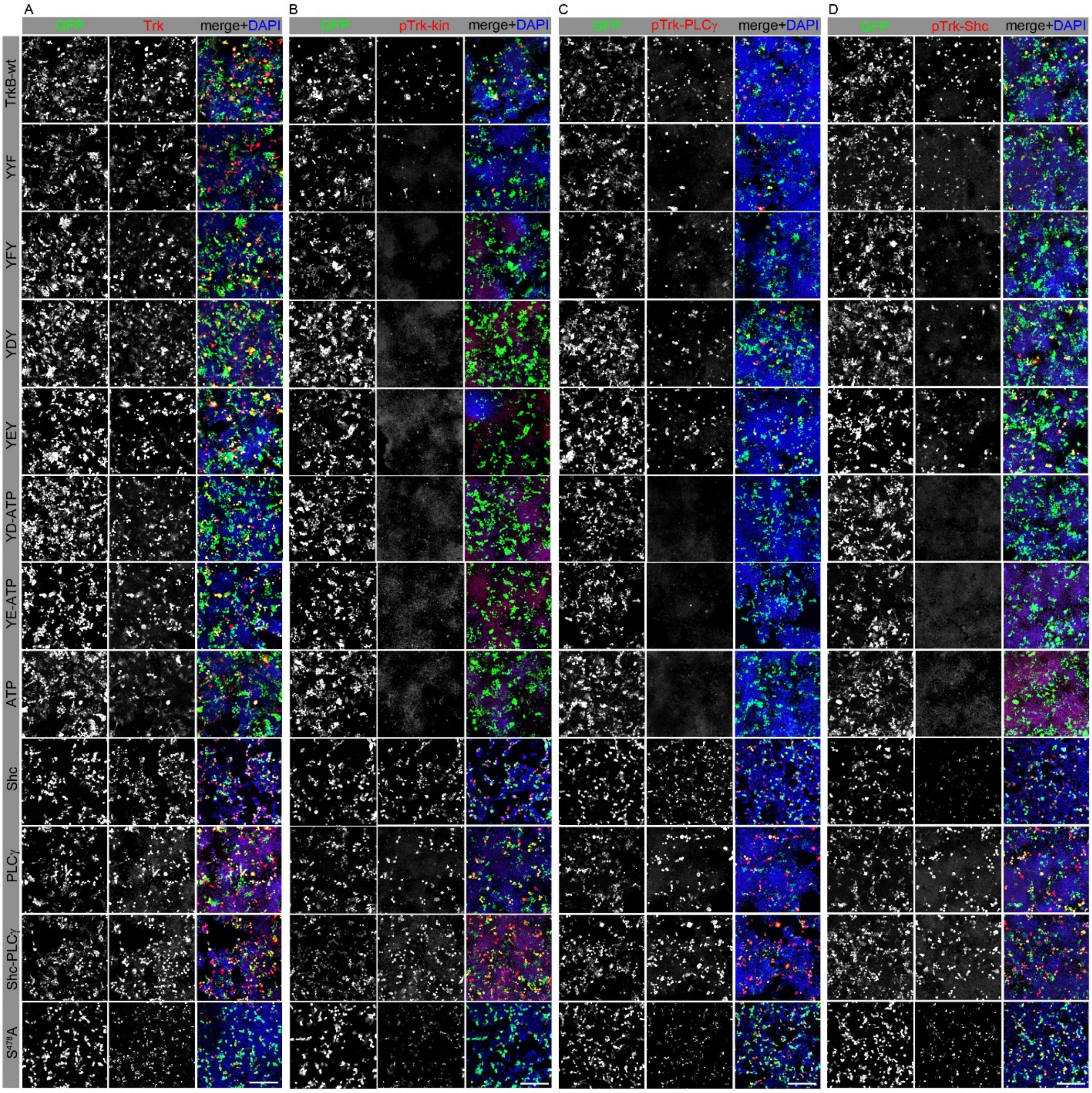

Fig. S2. Constitutive activation of TrkB by overexpression and immunoreactivity profiles of anti-Trk, anti-pTrk-kin, anti-pTrk-PLCγ and anti-pTrk-Shc for various TrkB mutants. A. Immunostaining of HEK293 cells expressing either TrkB wildtype or indicated TrkB mutants. Cells were co-transfected with GFP. Immunofluorescence of GFP (green) and panTrk (red, anti-panTrk (C-term) – A7H6R), together with DAPI as nuclear counterstain (blue). Cells were immunostained after an expression time of 48 h. panTrk binds to the C-terminus of the receptor and shows there the integrity of the open reading frame of all mutants. HEK293 themselves do not express TrkB. Confocal images; scale bar: 100 µm. **B.** Immunofluorescence of GFP (green) and pTrk-kin (red, anti-pY674/675-TrkA (anti-pY706/707–TrkB) C50F3), together with DAPI (blue). pTrk-kin antibody binds to the phosphorylated 2nd and 3rd Y residues in the YxxxYY motif of the Trk kinase domain. When these sites are mutated ($Y^{705/706}$), there is no fluorescence seen as the antibody no longer recognizes them (i.e. - YYF, YFY, YEY, YDY). When the ATP site is mutated ($K^{571}N$), lack of ATP prevents TrkB autophosphorylation and the Y residues in the kinase domain remain unphosphorylated (ATP, YD-ATP). All missense mutations in Y^{705} for D, E, or F, interrupted the anti-pTrk-kin immunoreactivity TrkB. Mutations in the Shc ($Y^{515}F$) and

PLCγ ($Y^{816}F$) sites did not affect the phosphorylation of the YxxxYY residues and cells remain positive for pTrk-kin (Shc, PLCγ, Shc- PLCγ). Confocal images; scale bar: 100 µm. **C.** Immunofluorescence of GFP (green) and pTrk-PLCγ (red, anti-pY785-TrkA (anti-pY816-TrkB) C67C8), and a DAPI counterstain (blue). Confocal images; scale bar: 100 µm. In kinase-dead TrkB mutants (ATP, YD-ATP) TrkB remains unphosphorylated. This shows that the antibody is phospho-specific in TrkB. Constitutive phosphorylation is seen in all other mutants, albeit at different intensities. A directed missense mutation at the PLCγ-site ($Y^{816}F$) creates a phospho-independent, immunoreactive site after paraformaldehyde fixation (see also D for anti-pTrk-Shc). **D.** Cells were co-transfected with GFP. Immunofluorescence of GFP (green) and pTrk-Shc (red, anti-pY490-TrkA (anti-pY516-TrkB) C35G9), and a DAPI counterstain (blue). Cells were immunostained 48 h after transfection. Confocal images; scale bar: 100 µm. In kinase-dead TrkB mutants (ATP, YD-ATP) TrkB remains unphosphorylated. This shows that the antibody is phospho-specific in TrkB. Constitutive phosphorylation is seen in all other mutants, albeit at different intensities. A directed missense mutation at the Shc-site ($Y^{515}F$) creates a phospho-independent, immunoreactive site after paraformaldehyde fixation (see Shc and Shc-PLCγ double mutant).

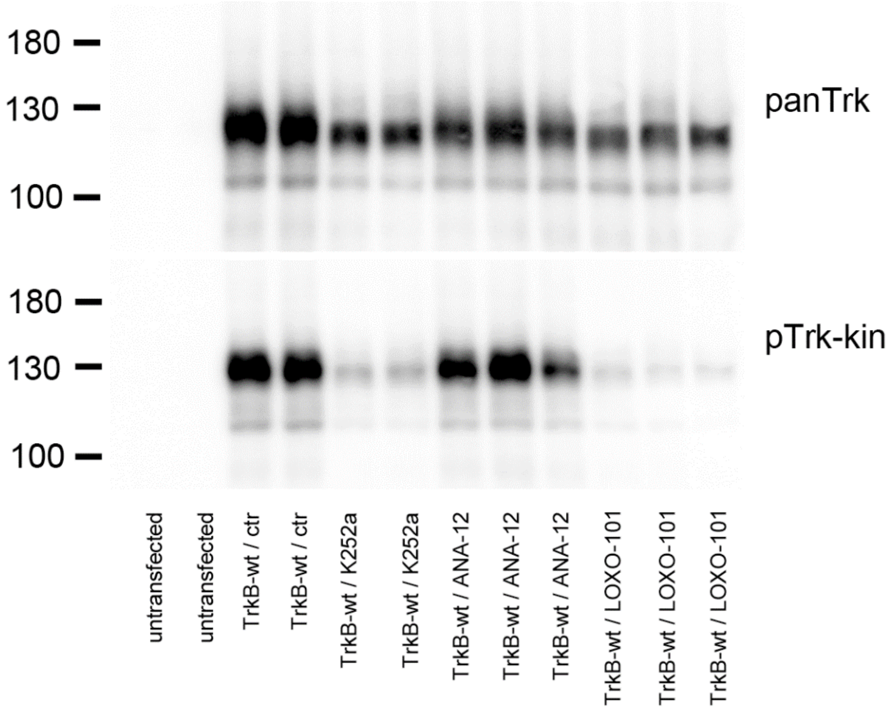

Fig. S3. ANA-12, a TrkB receptor domain inhibitor, does not inhibit TrkB self-activation. Western blotting of whole-cell lysates of HEK293 cells expressing TrkB. To inhibit ongoing TrkB kinase activity, cultures were preincubated with ANA-12 (10 µM), K252a (150 nM) or LOXO-101 (150 nM). DMSO served as solvent control. Blots were labeled with anti-TrkB-kin and anti-panTrk to detect overexpressed TrkB and to show lack of expression of TrkA or TrkC kinases in untransfected cells.

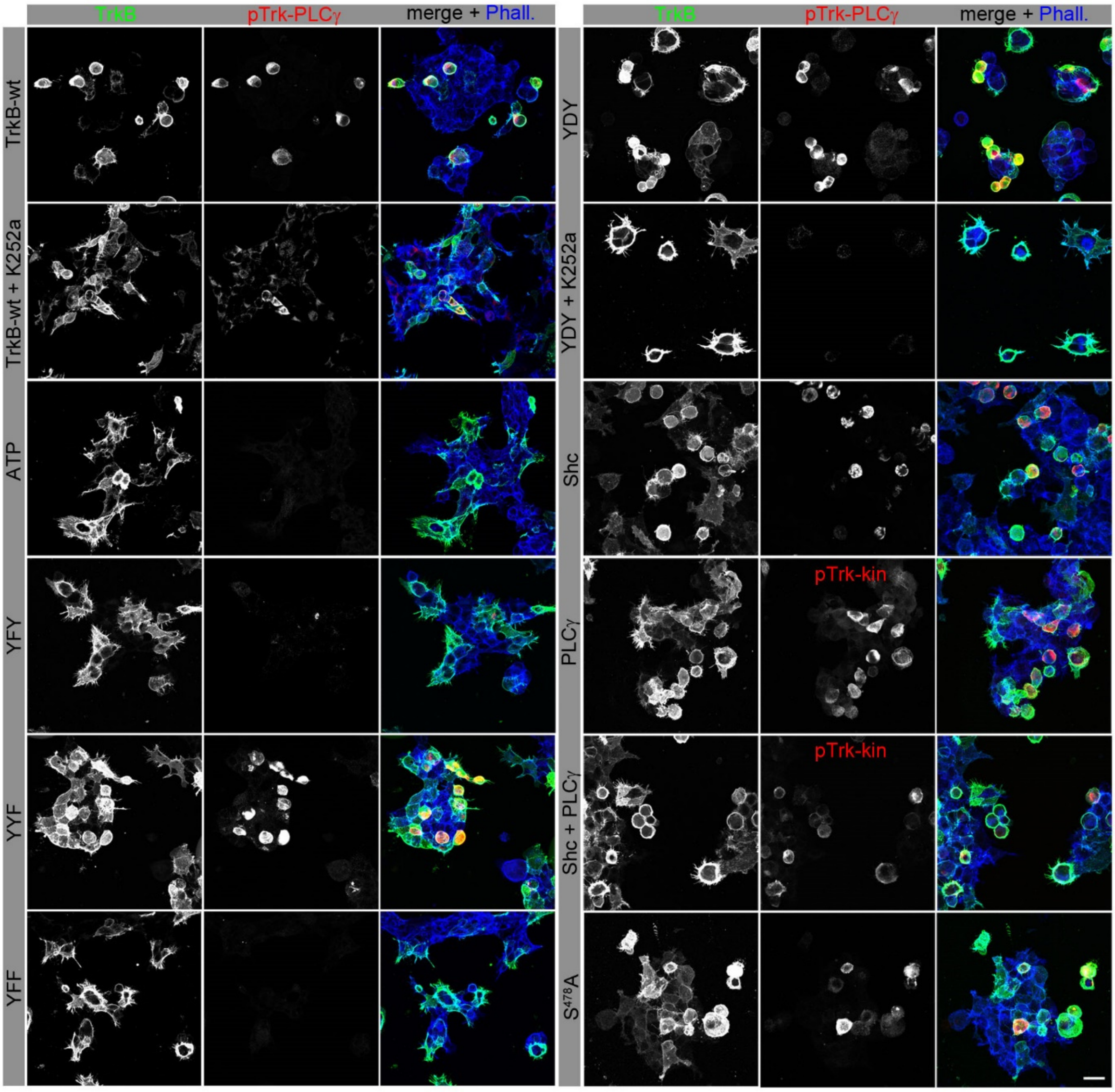

Fig. S4. Abundance-dependent auto-activation of TrkB kinase changes in actin morphology (supplementary for Figure 2F, G). Round-shaped cells express kinase-active TrkB mutants. Immunostaining of HEK293 cells expressing either TrkB wildtype or indicated TrkB mutants. Immunofluorescence of TrkB receptor (green) and pTrk-PLCγ (red). PLCγ ($Y^{816}F$) mutants (TrkB-PLCγ, TrkB-Shc-PLCγ) were stained with pTrk-kin (red). F-actin was labelled with Acti-stain-670 phalloidin (blue). Typically, filamentous cells express the kinase-dead ATP mutant of TrkB or the YxxxYY mutants (YFY and YFF). Treatment with 150 nM K252a for 30 min reverses the round shape of cells expressing TrkB wt or TrkB-YDY. Confocal images; scale bar: 25 µm.

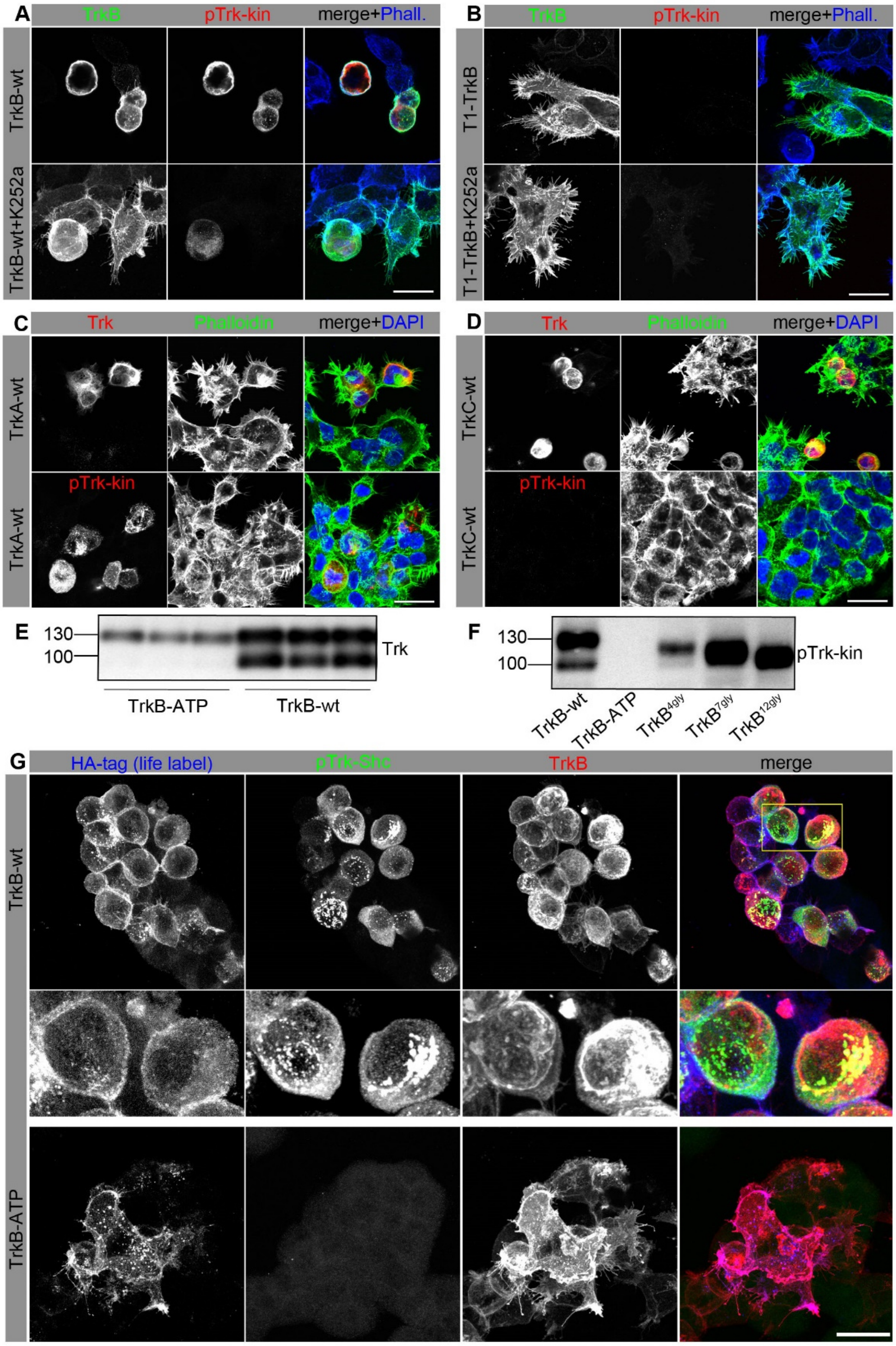

Fig. S5. A-D: Filopodia formation is preserved in the kinase deficient TrkB-T1 expressing cells, but not in Trk kinase expressing cells. A, B. TrkB overexpression causes changes in actin morphology of HEK293 cells. This effect can acutely be reversed by the Trk inhibitor K252a. HEK293 cells expressing the kinase-deficient, truncated slice variant TrkB-T1 remain phospho-inactive and filamentous for control and K252a-treated conditions. Immunofluorescence of

TrkB receptor (green) and pTrk-kin (red). F-actin was labelled with Acti-stain-670 phalloidin (blue). Confocal images; scale bar: 25 µm. **C, D.** TrkA and TrkC kinase overexpression leads to roundish cells. Immunofluorescence of panTrk receptor or pTrk-kin (red). Note that TrkC autophosphorylation is not detected by anti-pTrk-kin. F-actin was labelled with Acti-stain-670 phalloidin (blue). Confocal images; scale bar: 25 µm. **E-G: Intracellular localization and delayed glycosylation of constitutive active TrkB. E.** Western blotting of whole-cell lysates generated from HEK293 cells expressing TrkB-wt or the kinase dead mutant TrkB-ATP. After transient transfection, TrkB was expressed for 30 h. Lysates were probed with anti-panTrk, an antibody that detects TrkB at the intracellular C-terminus. TrkB-ATP shows a Mr of about 130 kDa, while TrkB-wt also runs at 90 kDa, a western blotting band typical for immature TrkB. **F.** Western blotting of whole-cell lysates generated from HEK293 cells expressing TrkB-wt, the kinase dead mutant TrkB-ATP or the TrkB-glycosylation mutants. After transient transfection, TrkB was expressed for 30 h. Lysates were probed with anti-pTrk-kin. All mutants are self-activated except for the kinase dead ATP mutant. The glycosylation mutants appear at different heights depending on the number of mutated glycosylation sites. **G.** Life labelling of TrkB-wt and TrkB-ATP. Cells expressing TrkB were life-labelled via an extracellular HA-tag for 15 min. Then cells were fixed and post-labelled with pTrk and anti-TrkB. Note accumulation of pTrk at intracellular, perinuclear sites and in vesicular clusters.

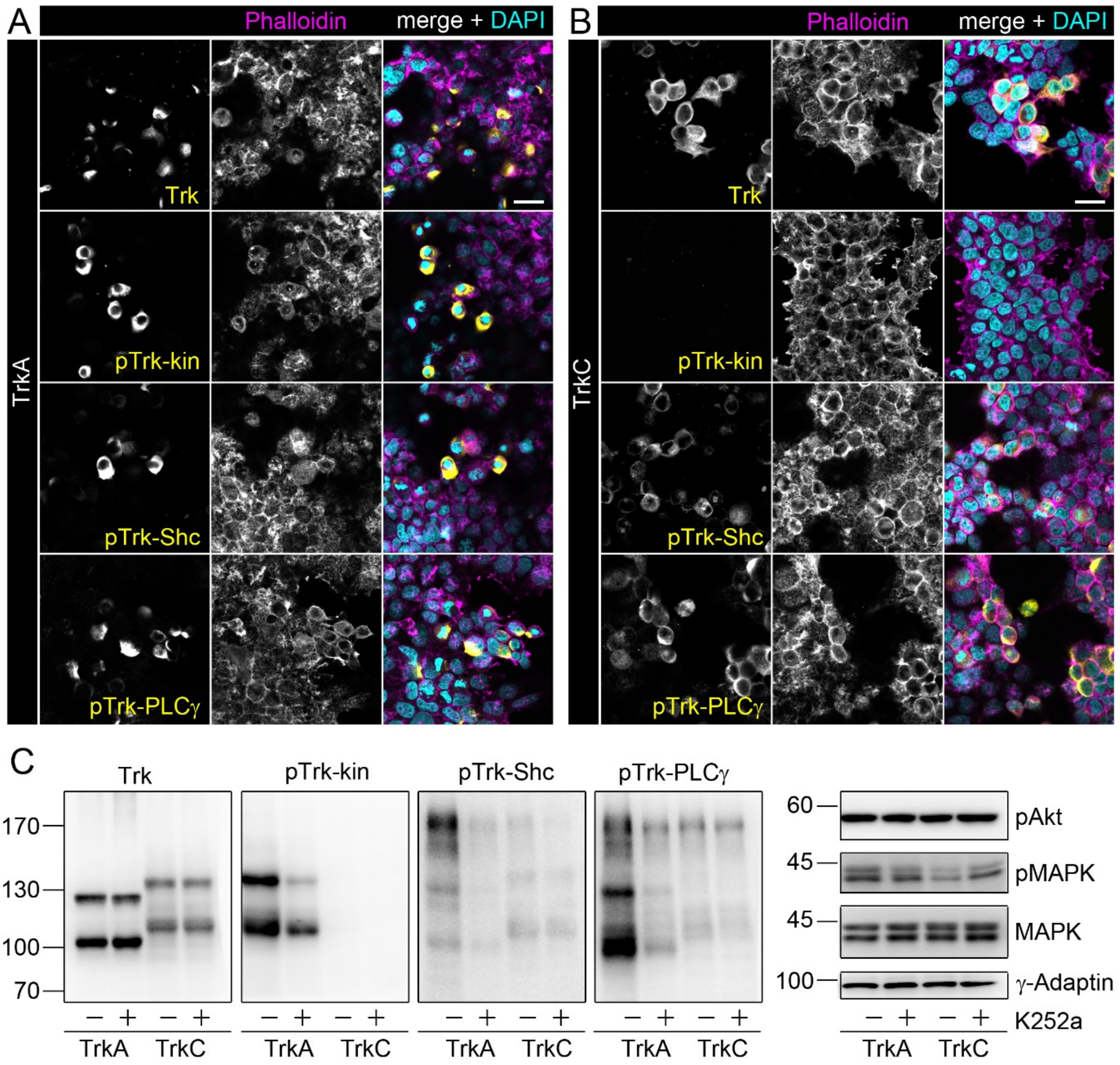

Fig. S6. TrkA and TrkC kinase overexpression leads to roundish cells. A, B. Immunofluorescence of panTrk receptor, pTrk-kin, pTrk-Shc, and pTrk-PLC⍰ (yellow) in HEK293 cells expressing either TrkA (in A) or TrkB (in B). Note that TrkC autophosphorylation is not detected by anti-pTrk-kin, but by anti-Trk-Shc and anti-Trk-PLC⍰. F-actin was labelled with Acti-stain-670 phalloidin (magenta). Confocal images; scale bar: 25 µm. **C.** Western blot analysis of cell lysates from HEK293 cell overexpressing either TrkA or TrkC. K252a (+) or solvent control (DMSO, -) were applied for 30 min. Antibodies are indicated. The figure shows that a combination of anti-pTrk antibodies used by immunofluorescence is suited to describe constitutive activation of TrkA and TrkC. Western blot results are less convincing.

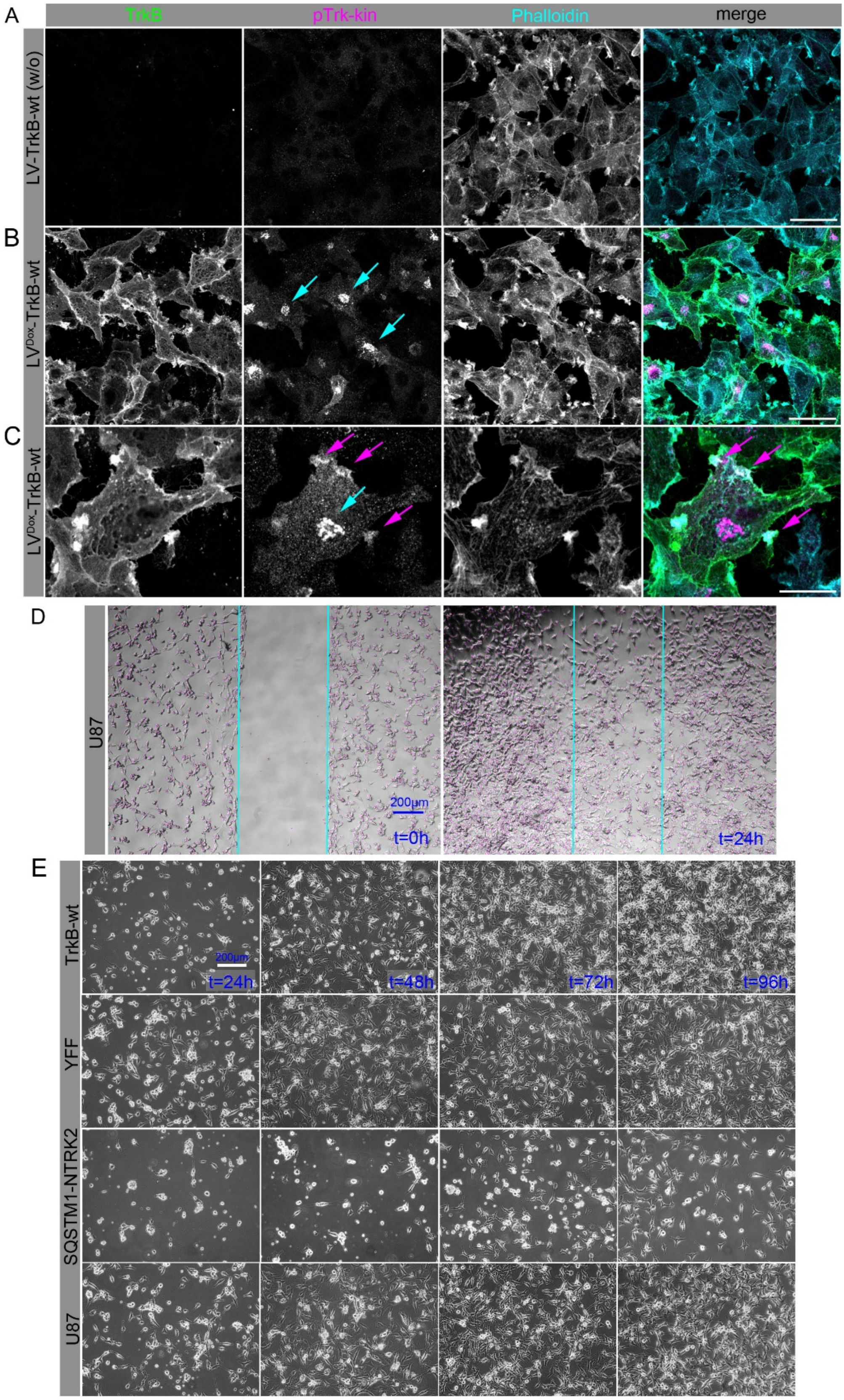

A
TrkB
pTrk-kin
Phalloidin
merge
LV-TrkB-wt (w/o)
B
LV^Dox-TrkB-wt
C
LV^Dox-TrkB-wt
D
U87
200µm
t=0h
t=24h
E
TrkB-wt
YFF
SQSTM1-NTRK2
U87
200µm
t=24h
t=48h
t=72h
t=96h

148

Fig. S7. U87MG cells expressing Trk-kinase constructs. A-C. Constitutive active TrkB localizes to the perinuclear Golgi-like region and accumulates in actin-rich protrusions of U87MG cells. Immunofluorescence of TrkB receptor (green), pTrk-kin (magenta), and Acti-stain-670 phalloidin (cyan). (in A) In absence of Doxycyline, TrkB expression is not detectable. (in B) Induction of TrkB expression with Doxycycline leads to constitutive activation of TrkB. pTrk signals are pronounced at the perinuclear, Golgi apparatus-like region (cyan arrows). Confocal image; scale bar: 50 µm. (in C) Confocal stack. pTrk-kin localizes also to F-actin rich protrusions (arrows in magenta). Scale bar: 10 µm. **D.** Migration of U87MG cells. Representative phase contrast microscopy images. Indicated in magenta are cells that were automatically counted by unbiased cell counting with ImageJ (see Material and methods). **E.** U87MG cells expressing TrkB-wt or SQSTM1-NTRK2 were not dying within the indicated time span of 96 hours, albeit the cells express a rather high amount of intracellular Trk kinase activity (Figure 5).

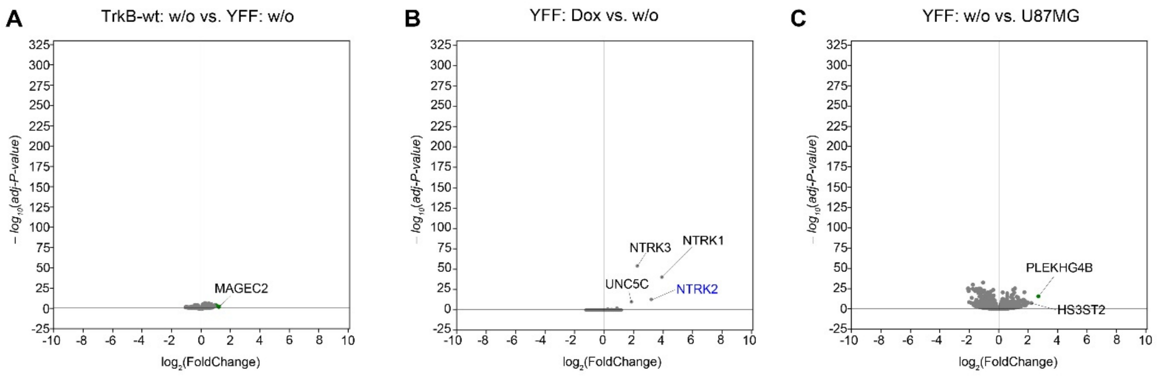

Fig. S8 TrkB in U87MG – RNA seq controls. A-C. RNA-seq analysis: Volcano plots showing gene expression patterns for various RNA-seq controls. Log2-fold-change values are plotted to P-adjusted values obtained from DESeq2 analysis of the transcriptome. (in A) TrkB-wt uninduced control (w/o) is compared to TrkB-YFF uninduced control (w/o), showing no change in the transcriptome patterns and no leaky TrkB expression. (in B) TrkB-YFF induced (Dox) when compared to uninduced control (w/o) shows predominantly an upregulation of the three NTRK genes as they share sequence homologies. Doxycycline induction causes expression of only NTRK and none of the immune response genes (as seen in Figure 6). Thus, lentiviral constructs themselves do not cause an anti-viral/immune responses in the cell line. (in C) TrkB-YFF uninduced control (w/o) is compared to the U87MG cell line, in order to eliminate possible underlying effects arising from the cell line or from the lentiviral infection itself (see also Figure 6F).

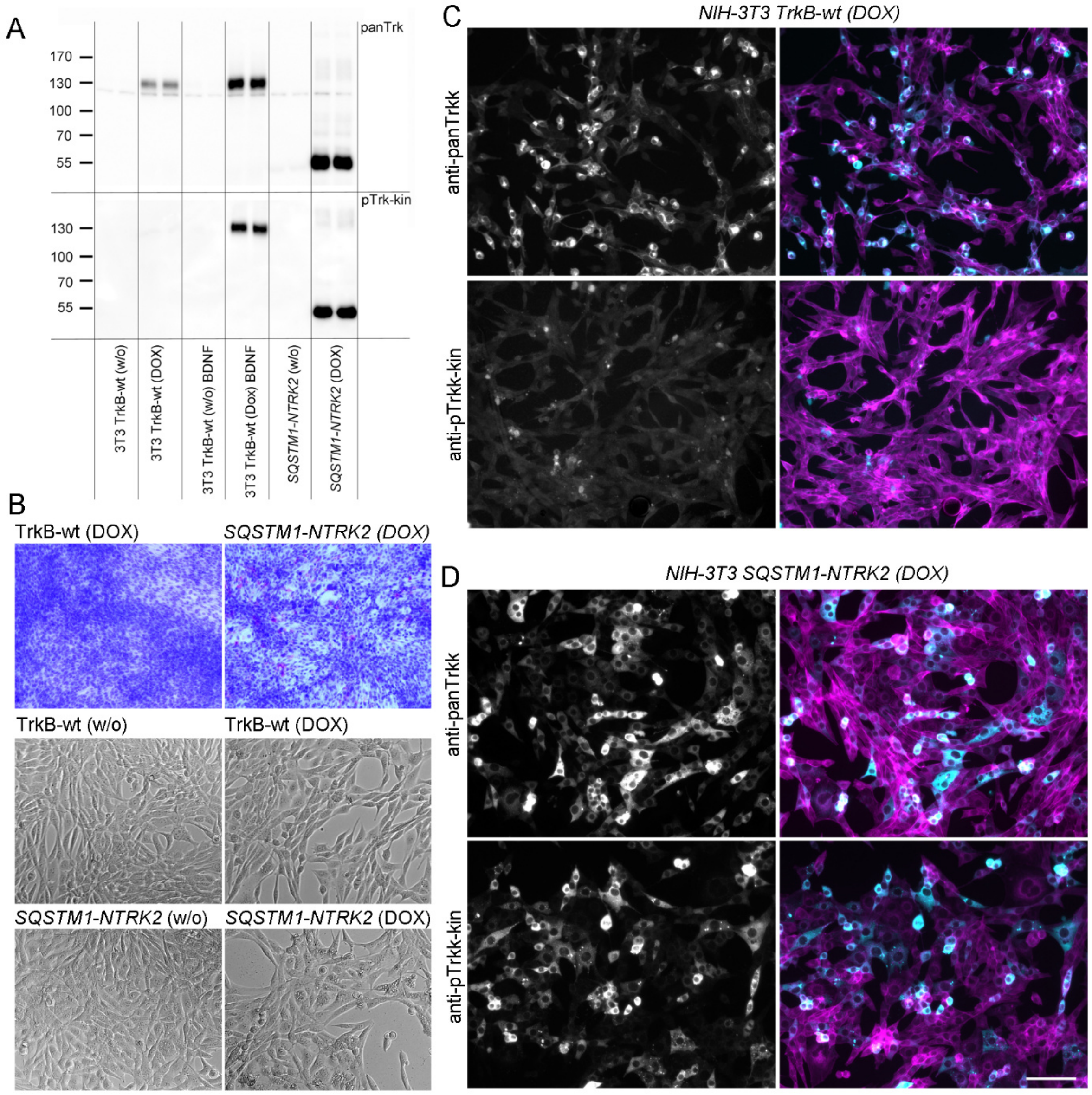

Fig. S9. NIH-3T3 cells expressing Trk-kinase constructs. A. Western blot analysis of cell lysates from NIH-3T3 cell lines expressing either TrkB-wt or SQSTM1-NTRK2. DOX was applied for 72 h, BDNF (20 ng/ml) was applied for 15 min. Cells were not serum-depleted. Antibodies are indicated. The figure shows that *SQSTM1-NTRK2*, but not TrkB-wt, becomes constitutive active in stable NIH-3T3 cells. TrkB-wt runs exclusively at 130 kDa in this cell model. Note the increase in the anti-panTrk signal by BDNF stimulation. **B.** Morphological cell transformation of NIH-3T3 cells by *SQSTM1-NTRK2*. *Upper layer:* Giemsa staining of confluent cell cultures expressing either TrkB-wt or SQSTM1-NTRK2. Note the change in culture morphology in *SQSTM1-NTRK2*-expressing cells. *Lower panels:* Brightfield image of indicated conditions, 72 days after expression induction with DOX. **C.** Immunofluorescence of TrkB-wt or *SQSTM1-NTRK2* in NIH-3T3 cells. Conditions and antibodies are indicated. Scale bar: 200 µm.

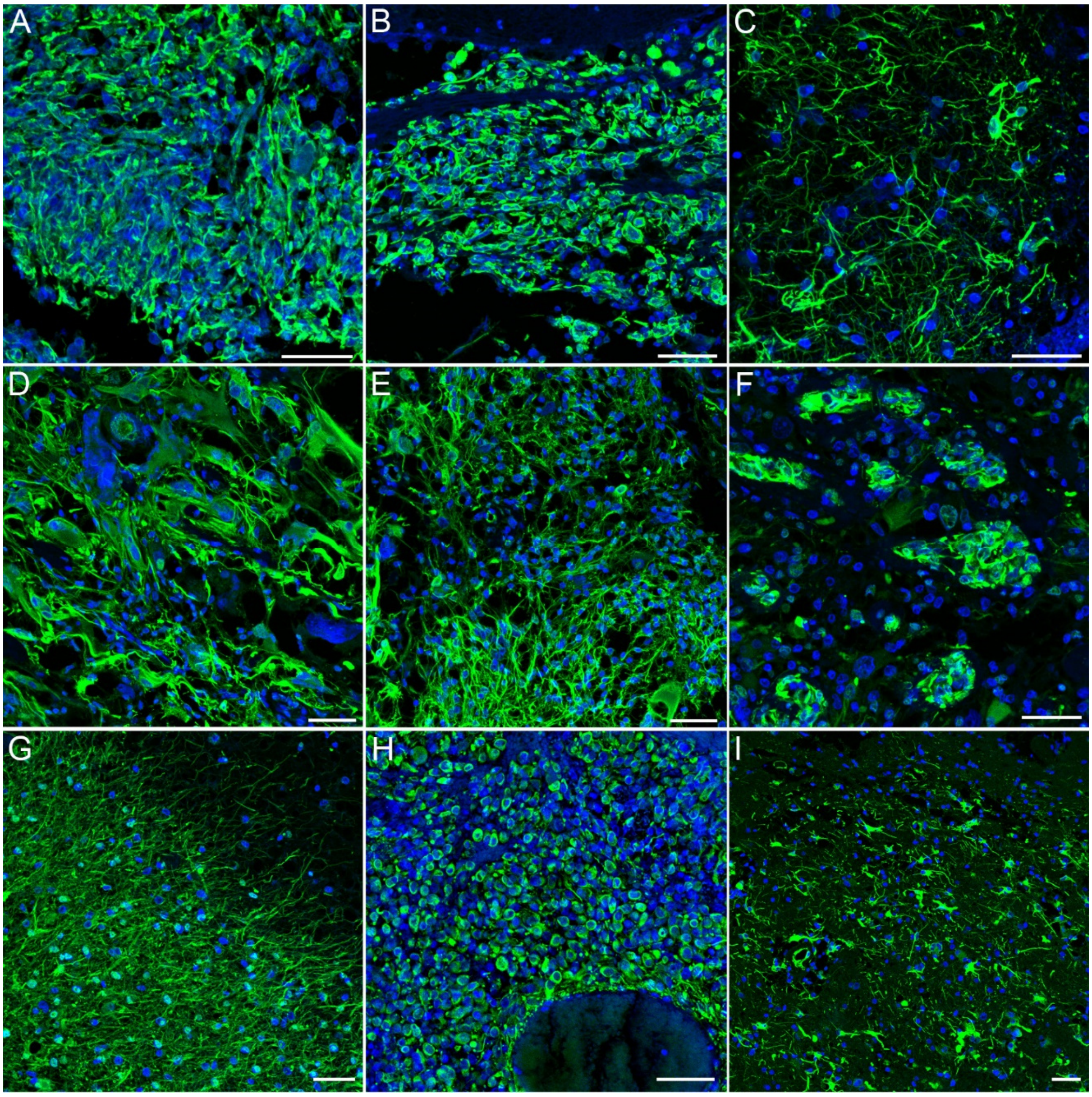

Fig. S10. Nestin immunoreactivity in WHO grade IV glioblastoma. A-I. Nestin (green) immunofluorescence signals in cryosections of post-mortem glioblastoma tissue. DAPI was used as nuclear counter stain (blue). Confocal images, maximum intensity projections, scale bar: 50 µm. In **A,B**: Different areas of the same patient sample. Nestin+ cells are more roundish and form a globular mass. In **C**: Neurite-like morphology of Nestin+ cells, In **D**: Diverse Nestin-morphologies ranging from cells with rather big somata, to smaller cells with disordered neurites. In **E**: Small spindle-like Nestin+ cells. In **F**: Individual Nestin+ cell clones. In **G**: Nestin+ cells ‘stretch’ their neurites in direction of a Nestin-negative area. In **H**: Small, Nestin-positive cells from a cell mass. The hole in the right lower part of the image shows an intratumoral haemorrhage full of erythrocytes. In **I**: Nestin+ cells show a disordered neuron-like morphology.
